# Epitranscriptomic profiling of VSMC phenotypes reveals uridine modifications linked to post-transcriptional regulation

**DOI:** 10.1016/j.bbrep.2026.102816

**Published:** 2026-09-28

**Authors:** Tobias Reinberger, Ayat Ismail, Torben Falk, Janina Fuß, Anja Wiechert, Elke Hammer, Tanja Zeller, Inken Wohlers

**Affiliations:** a University of Luebeck, Institute of Cardiogenetics, Germany; b University Hospital Schleswig-Holstein, University Heart Center Luebeck, Germany; c DZHK (German Centre for Cardiovascular Research), Partner Site North, Germany; d Biomolecular Data Science in Pneumology, Research Center Borstel, Leibniz Lung Center, Borstel, Germany; e Institute of Clinical Molecular Biology, Christian-Albrechts-University of Kiel, Kiel, Germany; f Department of Functional Genomics, Interfaculty Institute of Genetics and Functional Genomics, University Medicine Greifswald, Greifswald, Germany; g DZHK (German Centre for Cardiovascular Research), Greifswald, Germany; h University of Luebeck, Lübeck, Germany; i College of Medicine & Health Sciences, Khalifa University for Science and Technology, Abu Dhabi, United Arab Emirates; j Airway Research Center North (ARCN), German Center for Lung Research (DZL), Borstel, Germany

**Keywords:** Epitranscriptomics, RNA modifications, Vascular smooth muscle cells, Phenotypic transition, Post-transcriptional regulation

## Abstract

**Background:**

The phenotypic plasticity of vascular smooth muscle cells (VSMCs) can modulate atherosclerosis progression. Although several gene regulatory steps towards pro-inflammatory phenotypes have been well-studied, epitranscriptomic changes during this transition and their regulatory roles remain unexplored.

**Methods and results:**

Primary human VSMCs were stimulated with TGF-β1 to induce a contractile, matrix-producing state and with IL-1β plus PDGF-BB to induce a highly energetic, pro-inflammatory state, confirmed by Illumina bulk RNA sequencing and proteomics. Untargeted screening of mRNA base modifications using Oxford Nanopore Technologies direct RNA sequencing and xPore analysis revealed differential uridine modification within a GUUUU motif in pro-inflammatory VSMCs. Modified uridines were enriched in 3′-UTRs and at predicted RNA structural contexts consistent with accessibility, with implications for poly(A) tail dynamics and miRNA binding.

**Conclusions:**

Both TGF-β1 and PDGF-BB/IL-1β induce distinct epitranscriptomic landscapes composed of different modification types, often co-localized in the same transcript. Differentially modified uridines in mRNAs are abundant in a high-energy, pro-inflammatory VSMC state and are associated with post-transcriptional regulation. In summary, these findings suggest that U-centered nanopore signal changes are associated with post-transcriptional regulatory features during VSMC phenotypic transitions.

## Highlights

• Nanopore profiling reveals distinct RNA modification landscapes across VSMC states.
• RNA modification differences are enriched at uridines within GUUUU motifs.
• Differentially modified 3′-UTR uridines are enriched in predicted accessible RNA.
• Modifications overlap miRNA binding sites and correlate with poly(A) tail dynamics.

## Graphical abstract

**Figure:**
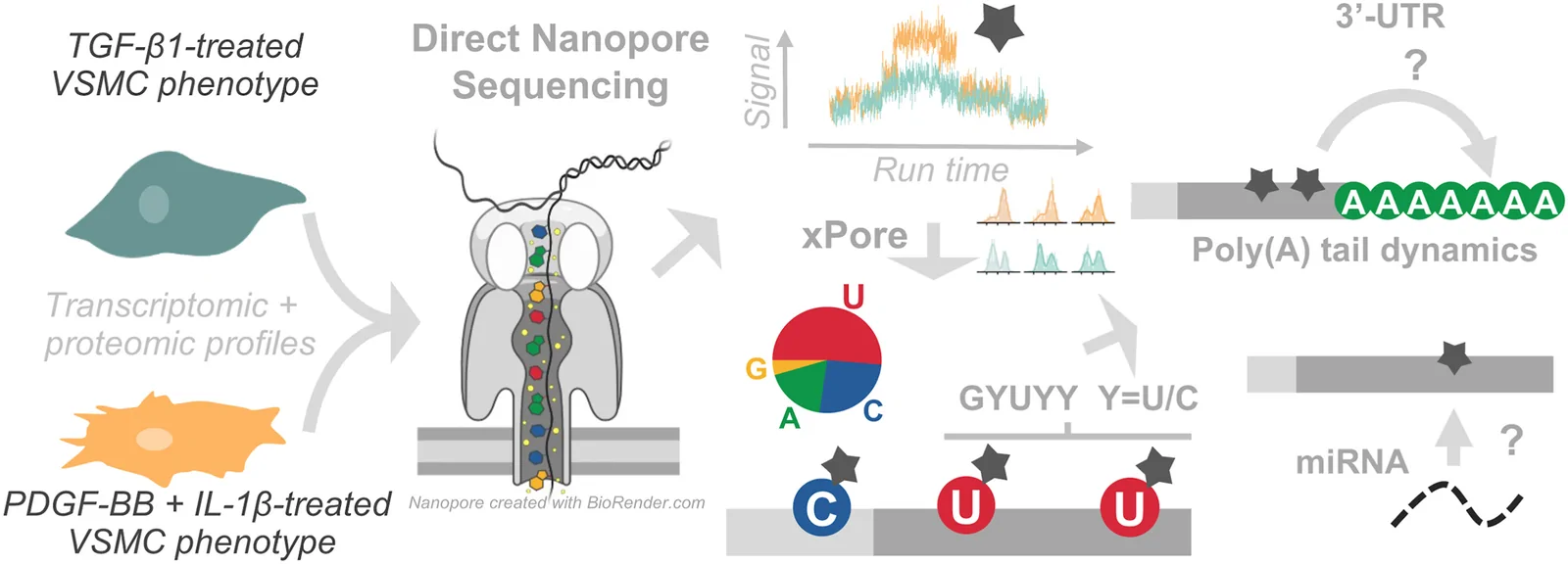

## 1 Introduction

Vascular cell adaptation within atherosclerotic plaques is one of the key features that modulates disease progression and can define the severity of atherosclerotic outcomes [1]. In particular, the immense plasticity of vascular smooth muscle cells (VSMCs) has been a key focus of research in cardiovascular disease over the last few years. While the role of cytokine-driven pathways, key transcription factors, epigenetic mechanisms, and non-coding RNAs in shaping VSMC phenotypic transitions is well described [[2], [3], [4], [5]], the influence of epitranscriptomic regulation remains largely unexplored and is only beginning to receive attention [6].

Epitranscriptomics characterizes and quantifies the collective set of chemical modifications of RNA. Previously, such modifications were investigated with techniques only being dedicated to specific modification types, such as N6-methyladenosine (m^6^A) and RNA types such as rRNA or tRNA [7]. With technological developments in third-generation sequencing, epitranscriptome-wide investigations are becoming feasible. As a result, direct RNA sequencing by Oxford Nanopore Technologies (ONT) allows the hypothesis-free identification of putative modification sites of any type by determining significant electric current differences between modified and unmodified bases, measured while RNA passes through a nanopore [8]. Following epitranscriptome-wide screens, chemical assignment of specific modification types can be performed and depends on model availability, sequencing chemistry, coverage, sequence context, as well as methods for orthogonal validation. More than 100 different types of RNA modifications have been described, and associations with many diseases have been reported [9]. Technology-fueled increased interest has resulted in a few initial enzyme- or modification-centered investigations in vascular disease being published within the past five years [10,11]. The importance of epitranscriptomic regulation in VSMC phenotypic transitions is in the early stages of being recognized. For example, platelet-derived growth factor (PDGF)-BB-induced adenosine-to-inosine (A-to-I) editing has been described as fostering the transition from the contractile, quiescent state to a synthetic, pro-inflammatory VSMC state [12]. Furthermore, it has been demonstrated that m^6^A mRNA methylation stabilizes transcripts of genes involved in VSMC-mediated vasoconstriction [13] and promotes VSMC proliferation, migration, and phenotypic switching [14], thereby highlighting the clinically relevant role of epitranscriptomic control in blood pressure and neointimal hyperplasia. Another disease-relevant RNA modification is 8-oxo-guanosine (8-oxoGuo), a product of RNA oxidation, which has been associated with atherosclerosis and reported as a predictor of mortality in individuals with type 2 diabetes [15]. Although the molecular consequences of RNA guanosine oxidation are well described in the context of cardiovascular diseases [16], the direct role in VSMC biology remains elusive. Nevertheless, oxidative stress is widely accepted to play a key role in chronic inflammation, to contribute to VSMC phenotypic switching [17], and to promote the development of unstable plaques [18].

Here, we employed hypothesis-free ONT direct RNA sequencing to extend the spectrum of VSMC-relevant RNA modifications beyond m^6^A and to obtain an unbiased view of the global epitranscriptomic landscape jointly for all modification types and across distinct VSMC phenotypic states. Inspired by a recent study in which ONT long-read cDNA sequencing was applied to investigate alternative splicing of contractile and proliferative, pro-inflammatory phenotypes, represented by transforming growth factor β1 (TGF-β1)- and PDGF-BB–treated human aortic smooth muscle cells [19], we treated VSMCs with TGF-β1 (condition C3). This VSMC phenotype was compared to pro-inflammatory VSMCs under PDGF-BB/interleukin 1β (IL-1β) treatment [20] (condition C4), and additionally to oxidative stress-prone VSMCs (C2) as well as oxidatively stressed VSMCs (C1). Here, we investigated whether epitranscriptomic alterations are associated with post-transcriptional regulatory features, including mRNA processing, poly(A) tail dynamics, miRNA-binding-site contexts, and protein output.

## 2 Methods

### 2.1 Cell culture and functional assays of vascular smooth muscle cells

Human primary vascular smooth muscle cells (VSMCs) were obtained from multiple commercial vendors (Thermo Fisher Scientific/Gibco, Cell Applications, Inc., and PromoCell) and two different vascular beds (aorta/artery and coronary). Vendors report donor/material procurement in accordance with the Declaration of Helsinki (see Table S1). VSMCs were cultured and expanded up to passage eight. Since VSMCs rapidly transition to a synthetic phenotype if seeded on a plastic surface, we cultured VSMCs on a collagen I hydrogel instead. In brief, cells were seeded onto collagen I hydrogels and allowed to attach for 24 h in medium containing 1% fetal bovine serum (FBS) and 1 μM cytochalasin D to inhibit contraction, which was critical to prevent SMC-mediated hydrogel contraction and to ensure an even VSMC monolayer for CellROX measurements.

In total, five VSMC phenotypes were generated using distinct treatment regimens, with all treatments performed in minimal medium containing 1% FBS. Irrespective of the treatment protocol, all VSMC phenotypes were harvested after a total culture period of six days. The experimental design was initially developed to investigate how oxidative stress influences VSMC phenotypic fate in the context of atherosclerosis, with a particular focus on mRNA oxidation. For the oxidatively stressed model (C1 phenotype), VSMCs were first exposed to 5 ng/mL TGF-β1 for four days to induce a contractile phenotype. To limit active contraction and contraction-associated cell detachment, 1 μM cytochalasin D was also added. A four-day culture period is sufficient for VSMCs to establish a matrix-producing phenotype, as previous studies have demonstrated substantial fibronectin and collagen I production in cultured VSMCs after 72 h [21,22]. Both are physiologically relevant components of the vascular extracellular matrix, providing structural support and integrin-mediated signalling cues to medial VSMCs [23]. The cells were then treated with 10 ng/mL PDGF-BB and 10 ng/mL IL-1β for two additional days to generate the C2 phenotype. These intermediate VSMCs were exposed to a high concentration of PDGF-BB (200 ng/mL) for 3 h to stimulate the endogenous production of superoxide anions and other reactive oxygen species as previously described [24]. A 3-h exposure was selected to allow the development of potential RNA oxidation while capturing early antioxidant gene-expression responses. This phenotype was designated condition C1 and was intended to reflect an oxidatively stressed state. To better understand the molecular basis of the increased susceptibility of C2 cells to oxidative stress, two additional phenotypes were generated for comparison. These conditions were included to identify gene-expression patterns and RNA modifications associated with either a contractile or pro-inflammatory VSMC state that might explain the oxidative-stress-prone phenotype of C2. To generate a stable contractile phenotype, VSMCs were treated with 5 ng/mL TGF-β1 and 1 μM cytochalasin D for four days and subsequently maintained for two days in minimal medium containing 1% FBS. This matrix-producing, contractile condition was designated the C3 phenotype. A pro-inflammatory phenotype was generated by culturing VSMCs in minimal medium containing 1% FBS for four days, followed by treatment with 10 ng/mL PDGF-BB and 10 ng/mL IL-1β for two days. This condition was designated the C4 phenotype. VSMCs maintained in minimal medium containing 1% FBS for the entire six-day period served as the untreated reference condition and were designated C5. These VSMC phenotypes were subsequently assessed using CellROX-based measurements of cellular oxidative stress and RNA sequencing. Detailed culture conditions and hydrogel preparation are provided in the Supplementary Material. A detailed description of functional assays such as oxidative stress assays, quantification of the mitochondrial membrane potential, and Seahorse assays is also provided in the Supplementary Material.

### 2.2 Short-read RNA sequencing

Short-read sequencing using the TruSeq stranded mRNA library preparation kit was performed for eight biological replicates (see Table S1; see Table S2 for read numbers and mapping rates) and five conditions: C1 VSMCs (TGF-β1 and PDGF-BB/IL-1β + PDGF boost), C2 VSMCs (TGF-β1 and PDGF-BB/IL-1β), C3 VSMCs (TGF-β1 only), C4 VSMCs (PDGF-BB/IL-1β only), and C5 VSMCs (untreated). RNA isolation and library preparation are described in the Supplementary Material. RNA sequencing (RNA-Seq) datasets were analyzed using a community workflow from the snakemake workflow catalog (https://github.com/snakemake-workflows/rna-seq-kallisto-sleuth) version 2.8.4. Following the workflow's and sleuth's default, differential expression was tested in sleuth using a likelihood ratio test with the full model including condition and cell line and a reduced model including only cell line. Thus, cell line was included as a covariate in the model to correct for cell line- and donor-specific effects. Mapping was performed against Ensembl cDNA sequences of Ensembl version 113. A detailed description of the workflow tools, which cover the enrichment analysis and isoform switch analysis with IsoformSwitchAnalyzeR [25] is provided in the Supplementary Material. Principal component analysis (PCA) was performed for the 1000 most variable genes after selecting the most highly expressed transcript per gene and keeping only transcripts with a minimum normalized log_2_ expression of 6. Gene set enrichment analysis was conducted using Enrichr [26]. The gene-set libraries “MSigDB Hallmark 2020”, “KEGG 2021 Human”, “Reactome 2022″, and “GO Biological Process 2023″ were used. Enrichr top-ranked pathways with more than seven overlapping genes are displayed.

### 2.3 Long-read direct RNA sequencing by Oxford Nanopore Technologies

Stimulated VSMCs cultured on Col I hydrogels were subjected to direct RNA-Seq using Oxford Nanopore Technologies (ONT) (Table S1; see Table S3 for read numbers and mapping rates) under four conditions: C1, C2, C3, and C4 (see above). To trigger endogenous ROS production, VSMCs were incubated with 400 ng/mL PDGF-BB for 3 h. RNA isolation was performed as described above for short-read RNA-Seq. Approximately 10 × 10^6^ stimulated cells per condition were used as input for direct RNA-Seq. Library preparation and direct RNA-Seq were conducted according to the ONT protocol SQK-RNA002. About 150 – 200 ng rRNA-depleted RNA was sequenced for 18 – 22 h using a GridION device with FLO-MIN106 flow cells with more than 1000 active pores. ONT direct RNA-Seq data were processed using our own snakemake workflow (described in detail in the Supplementary Material and available at https://github.com/iwohlers/2025_ont_rnaseq_vsmc), with tools installed in a reproducible fashion via conda using environment files provided as part of the workflow. The workflow uses guppy version 6.3.2 for basecalling and pycoQC [27] for quality control. Transcript expression levels were determined via mapping to cDNA sequences of Ensembl version 113 using nanocount [27] (transcript annotation from Ensembl v113). For differential modification detection, reads were mapped to transcript reference sequences (Ensembl v113) using minimap2 [28], followed by xPore [8] (version 2.1) used with nanopolish eventalign output, *i.e.*, modification positions refer to transcript coordinates. The xPore tool was run with three technical replicates (from a single biological replicate) per condition using the default settings, including a minimum coverage threshold of 15 reads per site. In brief, for each transcript position and associated 5-mer, xPore jointly models read-level current signals from all included samples (from both conditions) using a shared two-Gaussian mixture model. After fitting, the Gaussian component whose mean is closest to the canonical signal mean (see https://github.com/GoekeLab/x-pore/blob/master/xpore/diffmod/RNA002_5mer_model.csv) of the corresponding 5-mer is assigned as the unmodified component, whereas the other is interpreted as modification-associated. Replicate-specific mixture weights quantify the proportion of reads assigned to the modified signal component. Post-processing was performed using a dedicated script provided with xPore, retaining, for each k-mer, the site corresponding to the higher or lower mode, depending on which was more abundant. Only sites with a minimum coverage of 15 reads across all samples were retained for downstream analysis. Multiple-testing correction was performed using the Benjamini–Hochberg method. Sites significant at a false discovery rate (FDR) of 5% and exhibiting a differential modification rate (i.e., the difference in mean modification rate between conditions) greater than 25% were subsequently considered high-confidence differential modification rate (DMR) sites. Accordingly, an absolute DMR threshold of 25% was selected to focus on robust modification changes with larger effect sizes and potential biological relevance. Notably, this threshold represents the minimum differential modification level assessed in the original xPore benchmarking experiments [8]. Poly(A) tail lengths were estimated using nanopolish mode ‘polya’ (coverage>10). Coordinates of untranslated regions (UTRs) and coding sequences (CDS) were obtained using pybiomart v0.2.0. Normalization of nanocount counts and testing of differential expression were performed with DESeq2 [29], and an additional variance stabilization using rlog transformation was performed before principal component analysis, both as previously described for nanocount expression data [27]. These downstream analyses and visualizations are available as part of the FAIRDOMHub under https://fairdomhub.org/projects/461. Generation of xPore motif plots and estimation of poly(A) tail length can be found in the Supplementary Material. Due to the limited number of biological replicates, statistical comparisons of poly(A) tail lengths were performed at the transcript-observation level and should therefore be considered exploratory, as transcript measurements originating from the same biological replicate were not explicitly modeled as repeated observations.

### 2.4 Transcriptome-wide prediction of RNA structure elements

RNA secondary structure prediction and analysis were performed in Python using ViennaRNA and Forgi packages. For each modified base or k-mer, overlapping 150-nt mRNA sequence windows were extracted from Ensembl annotations and shifted stepwise across the modification site. Centroid secondary structures and minimum free energies (MFE) were predicted for each window. Structural similarity was quantified from dot–bracket representations using a sequence alignment–based scoring approach and used to cluster centroid structures by hierarchical clustering. Representative structures were selected based on cluster size and energy characteristics, and the lowest-MFE centroid structures were retained for downstream analysis. Secondary structure elements were annotated using Forgi, and enrichment of structural elements within k-mers was assessed using hypergeometric and Fisher's exact tests (for details, see Supplementary Material). P values were adjusted for multiple testing using the Benjamini–Hochberg procedure.

### 2.5 Prediction of RNA structural elements involved in miRNA binding

Wide-window prediction (>350 nt) of RNA secondary structures of the candidate target transcript ITGB1-204 was conducted with RNAplfold (version 2.7.0). Alignment of the miRNA involved two steps: local alignment of the seed sequence and global upstream alignment of the residual miRNA sequence (for details see Supplementary Material).

### 2.6 Small RNA sequencing

For the identification of highly expressed miRNAs, small RNA sequencing was performed for two technical replicates for each of four conditions (C2 – C5) (n = 1 biological replicate per condition). Approximately 3.8 × 10^5^ cells/well (HAOSMC IV) were seeded in 6-well plates that were coated with a Col I hydrogel (1.5 mg/mL). VSMCs were stimulated as described above for short-read RNA-Seq and RNA isolation, library preparation, and data analysis are described in the Supplementary Material. We investigated whether modified bases were part of miRNA seed regions, for which we considered 7mer-m8 sites, *i.e.*, bases 2-8 of the miRNA need to match the reverse complement of the region covering the modified base. Seed regions were obtained from TargetScan [30]. Annotation of publication-supported miRNA-target interactions was obtained from mirTarBase [31].

### 2.7 Proteomics

Protein quantification in TGF-β1– and PDGF-BB/IL-1β–stimulated VSMCs was performed by label-free quantitative mass spectrometry (MS) with four technical replicates per condition (n = 1 biological replicate per condition). Proteins were extracted, quantified, enzymatically digested, and analyzed by HPLC–ESI–MS/MS using an Orbitrap mass spectrometer. MS data were processed and normalized with Spectronaut using a human UniProt reference database, and protein abundances were calculated as maxLFQ values. Unique and razor peptides were considered. In total, 4995 proteins were identified with a minimum of two peptides per protein. Statistical analysis was performed at the peptide level, and protein set enrichment analysis was conducted using Enrichr [26]. Detailed sample preparation, acquisition parameters, and analysis workflows are provided in the Supplementary Material. Proteins were classified as DMR-positive when their parent genes contained at least one DMR site across aggregated transcript isoforms. Enrichment of DMR-positive proteins among differentially abundant proteins (p.adj ≤ 0.05) was tested using a one-sided Fisher's exact test, and fold enrichment was calculated relative to the quantified protein background. To determine whether this association was independent of abundance- and transcript-related covariates, the likelihood of a protein being differentially abundant was additionally modeled by logistic regression with DMR status as the main predictor and transcript count, median transcript length, mean gene abundance, and mean protein abundance as covariates. Continuous predictors were z-standardized, incomplete observations were excluded, and HC3 heteroskedasticity-robust standard errors were used for inference.

## 3 Results

### 3.1 Energetic and transcriptomic profiling of VSMC phenotypes

To study transitions of vascular smooth muscle cells with respect to oxidative stress, inflammatory signaling, gene regulation, and epitranscriptomic changes, we established an *in vitro* cell culture model and performed energetic and global transcriptomic profiling of these VSMC phenotypes (Fig. 1, Fig. S1, Fig. S2, and Table S4). As previously described [32], activation of TGF-β signaling promoted a quiescent, contractile-like VSMC phenotype, accompanied by reduced mitochondrial activity (Fig. S1), higher levels of canonical VSMC contractile marker genes (*e.g., ACTA2*, *CNN1*, and *TAGLN*; cf. Fig. 1C, Fig. S2, and Table S5), and activation of genes involved in extracellular matrix (ECM) organization (Fig. 1C–E; Table S6). A mesenchymal-like, ECM-producing VSMC phenotype has been reported to contribute to fibrous cap formation in atherosclerotic plaques [2] - a protective layer that stabilizes fatty deposits in the vessel wall and helps prevent plaque rupture. Although functional readouts were only assessed in VSMCs from one donor, we considered this experimentally induced *in vitro* phenotype to represent a matrix-producing and contractile VSMC state, while acknowledging that it does not fully recapitulate the complexity of VSMC states *in vivo*. PCA of the most variably expressed genes showed the largest variation (PC1) between TGF-β1-treated (C3) as well as untreated VSMCs (C5) and PDGF-BB/IL-1β–treated VSMCs (C1/C2/C4) (Fig. 1B, Fig. S2). Of note, we added IL-1β as it has been shown to synergistically promote PDGF signaling [20]. PDGF-BB/IL-1β–treated VSMCs (C4) exhibited an energetic and pro-inflammatory signature, with enhanced mitochondrial and glycolytic activity as well as increased expression of genes involved in cytokine-induced signaling (Fig. S1 and Fig. 1). In particular, IL-17, TNF/NF-κB, and RAS signaling pathways have been shown to worsen atherosclerosis and accelerate plaque progression [33,34].

**Fig. 1:**
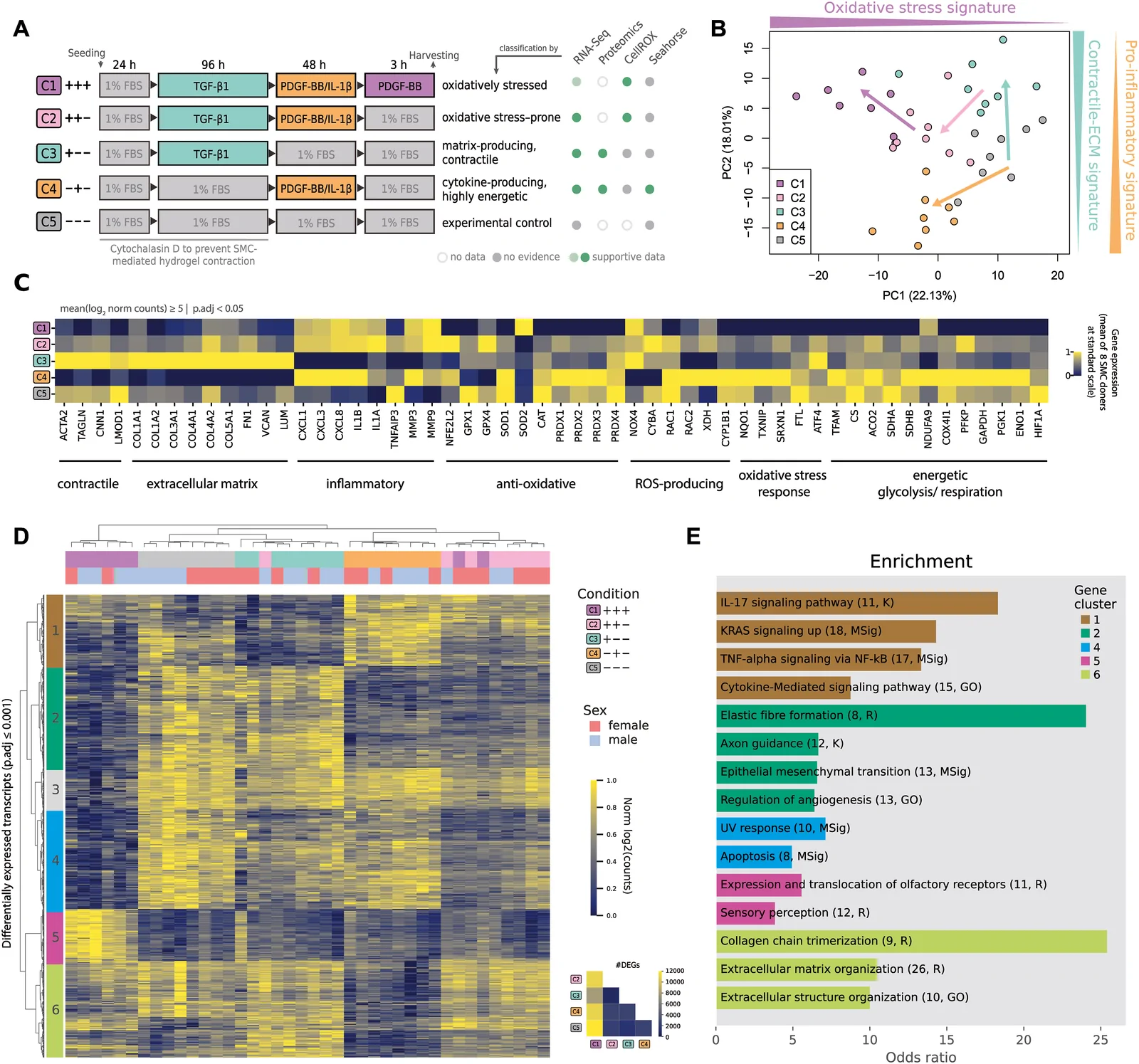
(**A**) Experimental design and definition of conditions C1–C5 used throughout the study. Each condition was sampled with eight biological replicates for short-read RNA-Seq, i.e., different VSMC cell lines. Classifications of VSMC subphenotypes are based on expression profiles of marker genes indicated in **C**, CellROX assays, or Seahorse assays shown in Fig. S1. (**B**) Principal component (PC) analysis of five conditions is based on the 1000 most variable transcripts with normalized and batch-corrected log_2_ expression ≥ 5. (**C**) Normalized expression levels of representative marker genes that are significantly differentially expressed after multiple testing correction across the five conditions for VSMC states. (**D**) Clustered heatmap showing row-scaled expression of the 1000 most variable canonical, differentially expressed transcripts (with FDR ≤ 0.001; mean log_2_ count ≥ 5) from the joint model across all conditions. (**E**) Up to four top-enriched pathways ranked by Enrichr for six gene clusters displaying condition-specific up- or down-regulation.

In addition, we created an intermediate VSMC state (TGF-β1 > PDGF-BB/IL-1β; C2) between C3 and C4 phenotypes, with mesenchymal transition–associated genes remaining inactive (cluster 2) while extracellular matrix and inflammatory genes are upregulated (clusters 1 and 6 in Fig. 1D). Notably, impairment of mesenchymal transition programs is often associated with reduced cellular adaptability to stress, as previously reported in the context of cancer [35]. Moreover, the concurrent production of ECM components and cytokines is energy-demanding and is therefore associated with increased ROS levels, which can promote mitochondrial dysfunction and overwhelm cellular antioxidant defenses. We therefore considered the intermediate VSMCs to be suggestive of an oxidative stress–prone state.

Finally, we added very high levels of PDGF-BB to this C2 VSMC phenotype characterized by elevated biosynthetic activity and reduced stress adaptation capacity, because high local concentrations of PDGF have been suggested in atherosclerotic lesions [36].

Indeed, hyperactivation of PDGF signaling resulted not only in elevated ROS levels (Fig. S1B), most likely through activation of mitochondria and NADPH oxidase as previously described [37], but also led to reduced expression of several genes essential for cell function, accompanied by a strong upregulation of olfactory receptor genes (Fig. 1D and E), which have been found to regulate pathophysiological processes in human airway SMCs and have been described as novel therapeutic targets [38].

In addition to pronounced transcriptional changes (Fig. S3), oxidative stress induced widespread differential splicing. A total of 1299 genes exhibited significant splicing differences between C3 VSMCs and remaining phenotypes (C1, C2, C4, C5), with *CCL20*, *CXCL8*, *FAH*, and *NOL4L* consistently affected across all comparisons (Table S7). Thereby, a detected splice isoform shift with a predicted coding potential was *CCL20*. Its increased expression in C4 VSMCs was accompanied by preferential usage of isoforms containing the chemokine interleukin-8-like domain (Fig. S4). Further, significant splicing differences were also detected in *ACTN1*, *TPM1*, and *FLNB* between contractile and oxidatively stressed phenotypes (C3 versus C1; Table S7), all of which have previously been reported as RBPMS-regulated smooth muscle cell splicing targets [39]. The involvement of these cytoskeletal genes is consistent with the extensive remodeling of actin and adhesion networks that accompanies vascular smooth muscle cell phenotypic switching.

### 3.2 Epitranscriptomic profiling of VSMC phenotypes

In order to investigate whether pro-inflammatory conditions accompanied by high levels of ROS can directly modulate mRNA base modifications, we used Oxford Nanopore (ONT) direct RNA sequencing and xPore (Fig. 2) to obtain a comprehensive overview of the RNA modification landscape. In general, up to 4% of mRNA bases of an individual transcript were found to be modified (*e.g.*, methylated; modification rate ≥ 0.5) in C3 VSMCs (Fig. 2B and Table S8). This proportion was higher in pro-inflammatory C4 VSMCs, with up to 6% of mRNA bases being modified (modification rate ≥ 0.5). While the absolute number of modified bases per transcript increases with transcript length, the ratio of modified bases per transcript correlates positively with ONT mRNA abundance but negatively with transcript length. Thus, shorter mRNAs tend to have a higher density of modified bases and to be expressed at higher levels. However, the correlation of abundance with the proportion of modified bases may simply reflect the increased likelihood of detecting modifications with higher abundances rather than a genuine biological trend. Given that only 6% of putative modified RNA bases appear to be guanine, we did not find evidence that the endogenous induction of oxidative stress was solely associated with exacerbated RNA oxidation, *e.g.*, represented by 8-oxoGu. Moreover, we found the lowest number of differentially modified mRNA sites (n = 384) when comparing oxidative stress-prone VSMCs (C2) with oxidatively stressed VSMCs (C1) (Fig. 2A and Table S11).

**Fig. 2:**
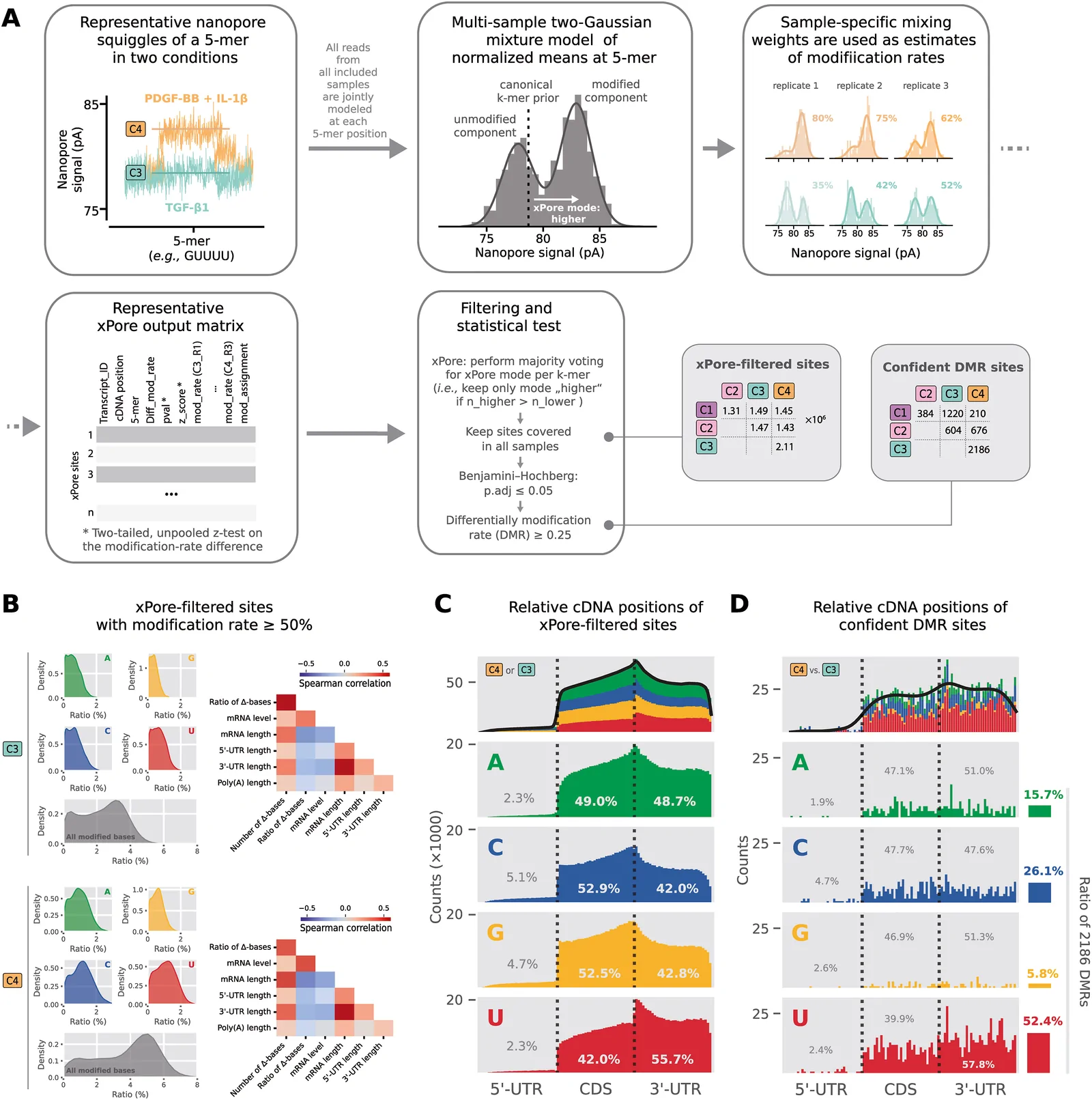
Detection of putative modification-associated signals by ONT direct RNA sequencing and xPore in vascular smooth muscle cells under conditions C1–C4 (n = 3 technical replicates and 1 biological replicate each) (**A**) Exemplary xPore workflow of VSMCs treated with TGF-β1 (C3) or PDGF-BB/IL-1β (C4). For each transcript position and 5-mer, xPore jointly fits read-level signals across all samples using a shared two-Gaussian mixture model. The component closest to the canonical 5-mer signal is assigned as unmodified (see Methods), and replicate-specific mixture weights estimate the modified-read fraction. In total, approximately 4 million transcriptomic positions (n) across 7568 transcripts were assessed with xPore. Differential modification was evaluated by comparing condition-specific modification rates using a two-tailed unpooled z-test. The number of xPore-filtered sites and high-confidence DMR sites after filtering are shown in the right panel for each comparison. (**B**) Proportion of modified (Δ) RNA bases (rate ≥ 0.5) in TGF-β1–treated VSMCs (C3) and PDGF-BB/IL-1β–treated VSMCs (C4). A Spearman correlation matrix is shown below. (**C**) The relative distribution of RNA bases (A, C, G, U) within UTRs and CDS is shown for all ∼2.1 million xPore-filtered sites detected in the comparison C4 vs. C3. (**D**) The relative distribution of 2186 sites with significant differential modification rates (DMRs) in UTRs and coding sequences (CDS) across 1430 transcripts.

However, comparison of C3 VSMCs and C4 VSMCs yielded the largest number of testable mRNA modification sites (2.1 million in C3 vs. C4), from which 5989 differentially modified RNA bases were identified after multiple-testing correction (FDR ≤ 0.05; Table S9). Subsequent filtering for a differential modification rate of at least 25% kept 2186 high-confidence differential modification rate (DMR) sites (Fig. 2A andTable S10). These DMR sites most likely reflect multiple modification types affecting adenosine, cytidine, and uridine (Fig. S5), which appear to be cytokine-dependent rather than stress-induced.

Across the pairwise comparisons, uridine-associated modifications were most prominent in VSMC conditions exposed to PDGF-BB/IL-1β, including C4 and C1 cells (TGF-β1 > PDGF-BB/IL-1β > PDGF-BB boost), suggesting a treatment-associated enrichment of uridine modification rather than an effect restricted to a single comparison. Consistent with this pattern, the motif plots in Fig. 3A (C4 vs. C3) and Suppl. Fig. S6 (C3 vs. C1, C2, and C4) show a higher number of uridine-centered modified sites in comparisons involving PDGF-BB/IL-1β–treated conditions. Uridine modifications often occurred in GY-U-YY motifs, with Y being a pyrimidine base (C or U) (Fig. 3A and B). Consequently, GUUUU was the most prevalent 5-mer context (209 DMR sites in 194 transcripts), showing ∼80-fold enrichment in C4 versus C3 VSMCs across all mRNA sequences analyzed with xPore (Fig. 3A). Affected genes were enriched for targets of the transcription factors TCF21, JUN (c-Jun), and JunD, as well as for pathways related to epithelial–mesenchymal transition, immune processes, cell migration, and PI3K/AKT/mTOR signaling (Table S12). Analysis with xPore revealed that many transcripts harbor multiple differentially modified sites (*e.g., POSTN* in Fig. 3C), and co-modification of uridine together with adenosine and/or cytidine was frequently observed (cf. Table S13). Overall, the comparison of C3 VSMCs and C4 VSMCs yielded 1258 genes expressing transcripts that contain one or more DMR sites, with the top affected genes being *FN1* (18 sites, 9 transcripts), *ITGB1* (15 sites, 12 transcripts), *HDLBP* (13 sites, 3 transcripts), and *TUBB* (10 sites, 5 transcripts) (Fig. 3D and Table S14; note that the more transcripts are linked to a gene, the greater the likelihood of detecting DMRs and the higher the DMR-to-transcript ratio after aggregating all transcripts). Other DMR-affected genes and transcripts involved in VSMC-relevant processes are highlighted in Table S15 and Fig. S7. Excluding *COL6A2* and *PXDC1* (Fig. S8A and S8B), none of these notably DMR-affected genes is among the 272 genes for which significant short read sequencing-based differential splicing was detected between C3 and C4 VSMCs (Table S7), suggesting that DMR sites may not affect alternative splicing.

**Fig. 3:**
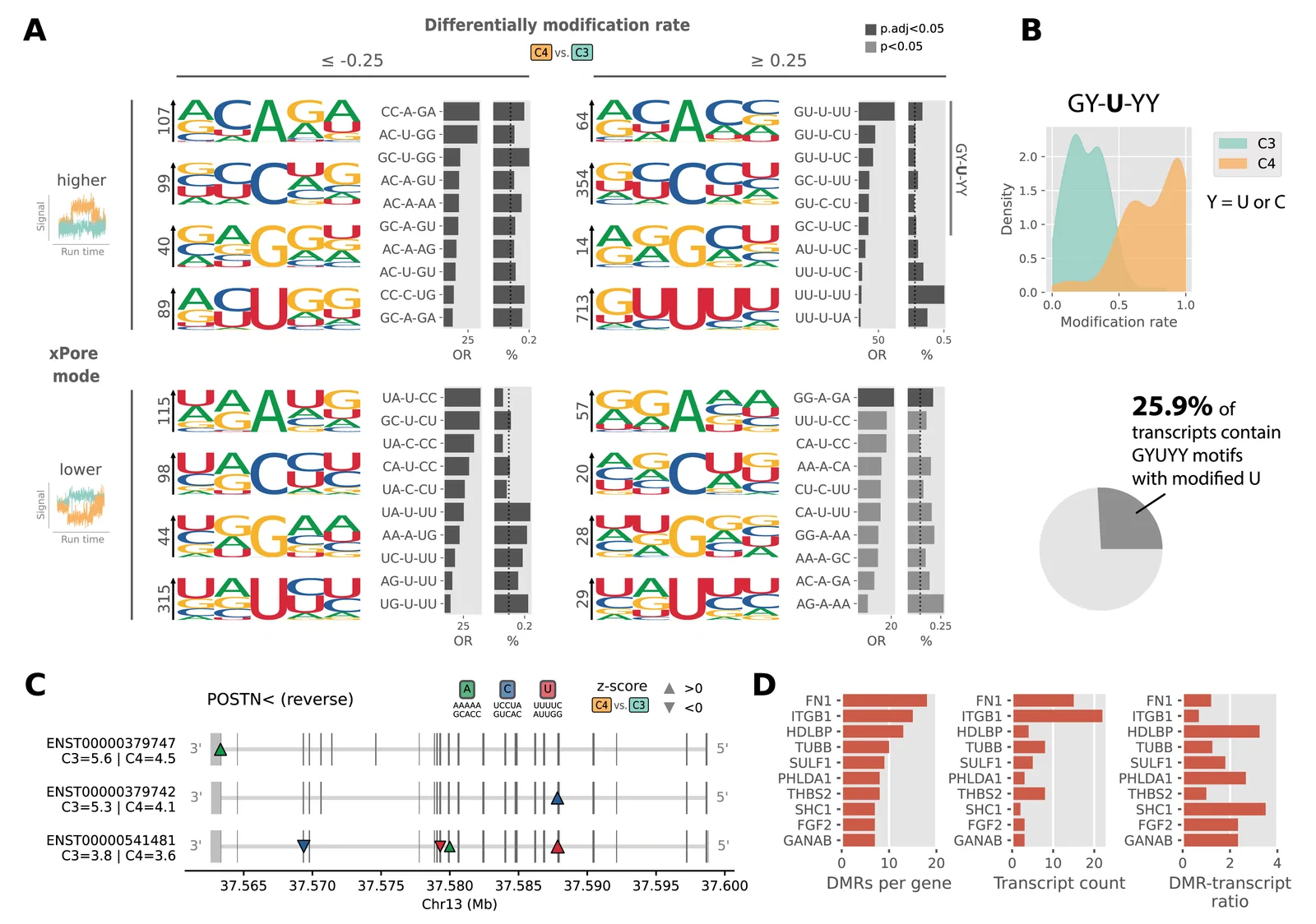
Sequence motif analysis of confident differential modification rate (DMR) sites between PDGF-BB/IL-1β–treated VSMCs (C4) and TGF-β1–treated VSMCs (C3). (**A**) Sequence motif analysis of confident DMR sites, stratified by differential modification rate (C4 vs. C3; ≤ −0.25 or ≥ 0.25) and xPore signal mode (“higher” or “lower”). Sequence logos show nucleotide frequencies at the five positions surrounding the putative modified site. The odds ratio (OR) and percentage (%) of the ten most abundant 5-mer motifs are shown adjacent to each logo. Dark and light bars indicate motifs passing the indicated statistical significance thresholds. Motif analysis identified U-rich sequences, including **GUUUU/GY-U-YY motifs**, among sites with increased modification rates in C4. The xPore “higher” mode denotes increased Nanopore current intensity at the putative modified base. (**B**) Distribution of modification rates for sites matching the **GY-U-YY** motif (Y=C or U) in C3 and C4. The accompanying pie chart shows the proportion of transcripts containing a GYUYY motif with a modified central uridine. **(C)** Transcript-level representation of differential modification sites in *POSTN*. Individual transcript isoforms and the positions of detected DMRs are shown together with the direction and magnitude of the C4-versus-C3 z-score. (**D**) Genes with the highest DMR burden, shown as the number of DMRs per gene, transcript count, and DMR-to-transcript ratio.

### 3.3 Association of base modification with protein abundance

Since we detected the largest number of DMR sites when comparing TGF-β1–treated C3 with PDGF-BB/IL-1β–treated C4 VSMCs, we first performed a differential expression analysis, resulting in 2306 differentially expressed genes (p.adj ≤ 0.05; cutoff of 1.5-fold change), of which about 5% were associated with at least one DMR site (Fig. 4A). We subsequently quantified the proteome of these VSMC phenotypes (Table S16), resulting in 4995 identified proteins with a minimum of two unique and razor peptides, of which 1171 were significantly differentially abundant after multiple testing correction (p.adj ≤ 0.05) (Table S17). At a cutoff of 1.5-fold change, 249 proteins were downregulated and 161 were upregulated in PDGF-BB/IL-1β–treated (C4) VSMCs compared to TGF-β1 treatment (C3), of which ∼30% and ∼20% were derived from genes with at least one DMR site, respectively (Fig. 4B). Enrichment analysis confirmed the pro-inflammatory signature of C4 VSMCs (Fig. 4A and B; Table S18). In order to investigate a potential effect of RNA modifications on protein levels, we focused on transcripts detected at a minimum of 10 transcripts per million (TPM) in both Illumina and ONT sequencing. At this threshold, log10-transformed RNA levels derived from Illumina and ONT correlate with a Pearson's correlation coefficient of r = 0.66 for C3 and r = 0.67 for C4 (cf. Fig. S9A), which is within the range reported previously [40]. In addition, transcript counts were aggregated per gene since proteomics is not isoform-sensitive. Protein levels aligned with their corresponding gene expression values in C3 VSMCs and C4 VSMCs where both were detectable (Fig. 4C and Fig. S9B). This relationship is independent of the presence of differentially modified RNA bases (DMR sites in C4 vs. C3). However, DMR sites are more frequently identified at higher gene/protein levels, which likely reflects both biological and technical factors, such as the fact that DMR sites are easier to detect at higher levels of abundance. Proteins expressed at higher levels are associated with higher gene expression and thus transcript sequencing coverage, resulting in disproportionately more sites being tested. Notably, the proportion of DMR-positive mRNAs decreases from approximately 22% among mRNAs with detectable protein to about 8% among those for which no corresponding protein was detected, despite comparable mRNA levels. Although potential effects of read coverage, transcript length, expression level, and protein detectability cannot be excluded, this observation raises the possibility that a subset of DMRs may be associated with translational activity. Among proteins/genes linked to more than two DMR sites, changes in transcript abundance were strongly mirrored at the protein level, reflected by a strong correlation (r = 0.7; p < 0.001) between the log_2_ fold change in gene expression and the corresponding log_2_ fold change in protein abundance (Fig. 4D and Fig. S10). Moreover, proteins and corresponding parent genes with more than two differentially modified mRNA bases across their aggregated transcripts were significantly enriched among differentially abundant proteins. Of 165 proteins meeting this modification threshold, 71 were differentially abundant (p.adj ≤ 0.05), compared with 1024 of 4129 proteins overall, corresponding to a 1.7-fold enrichment (one-sided Fisher's exact test, p < 0.001; cf. Fig. S10). This enrichment remained evident for proteins associated with at least one DMR site (Fig. S10). Consistent with this, DMR status (≥1 DMR site = 1; no DMR site = 0) remained significantly associated with differential protein abundance in an adjusted logistic regression model (with 1 = differential protein abundance; p.adj ≤ 0.05; and 0 = no significant difference) after accounting for transcript count, median transcript length, mean gene abundance and mean protein abundance. DMR-positive genes/proteins had 1.38-fold higher odds of being differentially abundant than DMR-negative genes/proteins (OR = 1.38, 95% CI 1.16–1.64, p = 2.5 × 10^−4^). As expected, protein abundance was the strongest covariate associated with the likelihood of differential protein abundance (OR = 1.44, p = 2.2 × 10^−15^), while transcript length and gene-level expression showed weak positive associations (OR = 1.15 and OR = 1.12, p < 0.05; respectively). Transcript count was not associated with absolute protein log_2_ fold changes. The overrepresentation of DMR-associated proteins in differentially abundant proteins is compatible with, but does not establish, a role for DMR sites in transcriptional programs that may also influence protein expression.

**Fig. 4:**
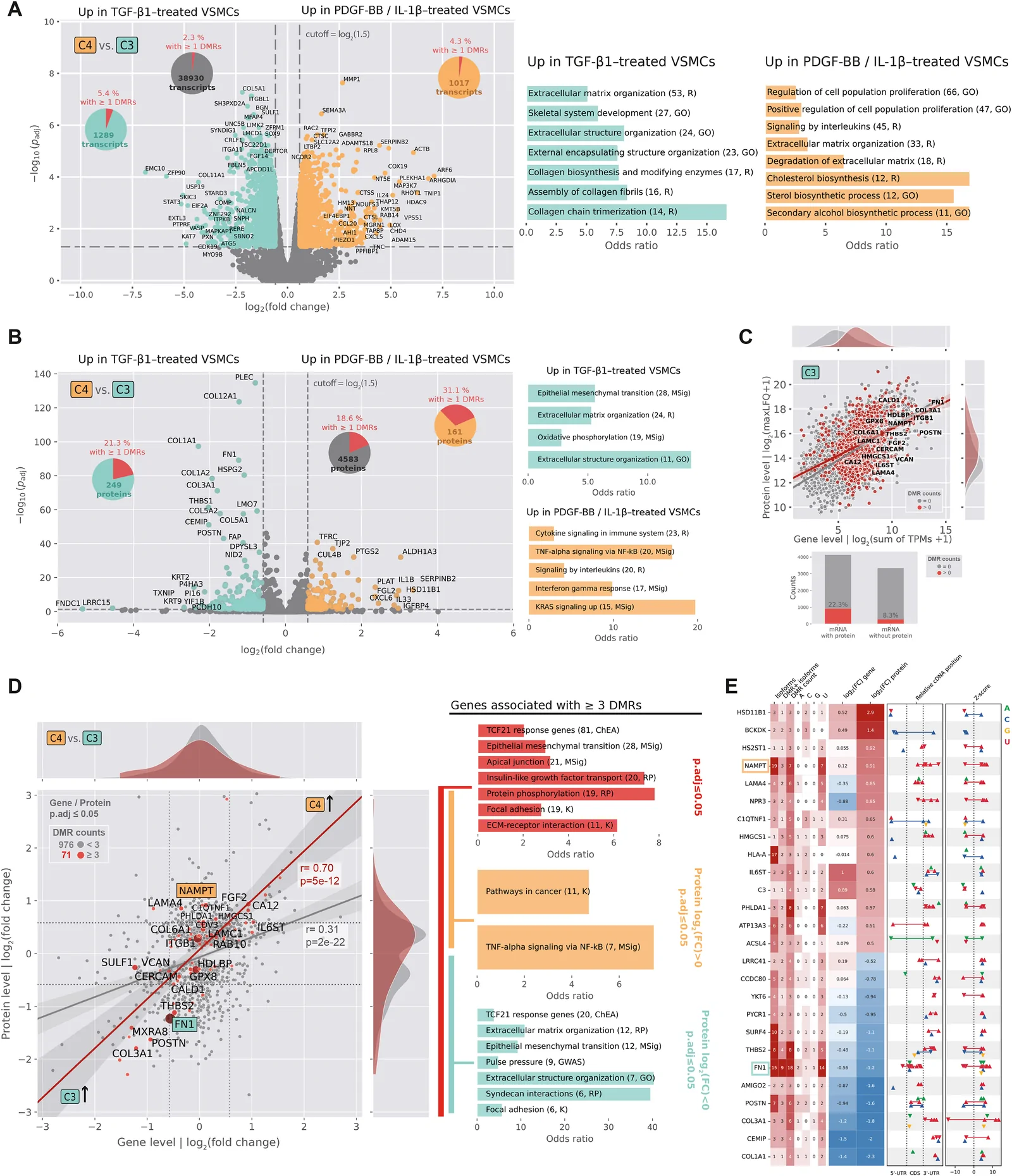
Association between differential modification rate (DMR) site burden and gene/protein abundance in vascular smooth muscle cells. (**A**) Differential gene expression analysis of VSMCs treated with TGF-β1 (C3) or PDGF-BB/IL-1β (C4). Transcript counts are from short-read RNA-Seq with n = 8 biological replicates per condition. (**B**) Differential proteomic analysis of C3 VSMCs and C4 VSMCs (n = 4 technical replicates and n = 1 biological replicate per condition; one VSMC line HAOSMC IV). Volcano plot and enrichment analyses highlight canonical TGF-β1– and PDGF-associated proteins and pathways (GO=Gene Ontology; MSig = Molecular Signature database; R=Reactome database). (**C**) Regression analysis of gene-level expression (aggregated TPM from long-read RNA-Seq) versus protein-level expression in C3 for transcripts with (red) or without (gray) differentially modified RNA bases (DMR sites; C3 vs. C4). Gene-level expression and protein abundance are derived from the same VSMC line HAOSMC IV. (**D**) Same analysis as (**C**) using log_2_ fold changes (C3 vs. C4). The Pearson correlation (r; significance assessed with *t*-test) was calculated for genes with ≥ 3 DMR sites across all isoforms and < 3 DMR sites. Gene set enrichment analysis was performed for genes with ≥ 3 DMRs and stratified by corresponding protein log_2_ fold change (>0 or < 0). (ChEA = ChIP-X enrichment analysis; GWAS = GWAS catalog; K=KEGG database; RP=Reactome Pathways). (**E**) Overview of genes with |log_2_FC| > 0.5 (FC = fold change), discordant gene–protein log_2_FC (>0.25), and > 2 DMR sites across all isoforms, including isoform counts, DMR site position in untranslated regions (UTR) or coding sequence (CDS), and z-scores (C4 vs. C3) with xPore mode as arrows (“higher” = up, “lower” = down).

Of note, differentially abundant proteins and corresponding genes were found to be enriched in TCF21 response genes in the matrix-producing, contractile phenotype C3 (Fig. 4D); TCF21 is a known driver of VSMC state transitions in atherosclerosis, with a role in VSMC switching to myofibroblast-like cells, and has been shown to have an atheroprotective effect [41]. Moreover, a subset of genes, including *FN1*, *POSTN*, *COL3A1*, and *CALD1* (see Fig. 4D–E), is associated with multiple DMR sites and downregulated in pro-inflammatory VSMCs (C4); all have established roles in VSMC biology associated with enhanced plaque stability or reduced atherosclerosis [5,42].

A complete overview of genes with notable differences in corresponding protein levels is shown in Fig. 4E. Neither the relative position of DMR sites within UTRs or coding sequences nor the direction of modification changes suggested a consistent rule governing DMR-dependent transcriptional or translational regulation (Fig. 4E). Because only one VSMC donor was included, with four technical replicates per condition (n = 1 biological replicate per condition), the generalizability of these findings is limited, and the results should be regarded as exploratory.

### 3.4 Transcriptomic landscape of mRNA modification–related genes

Since uridine was the most frequently modified RNA base, we first examined the expression level of pseudouridine synthase genes in TGF-β1–treated C3 and PDGF-BB/IL-1β–treated C4 VSMCs in a candidate screen of RNA modification enzymes. A notable increase in *PUS7* levels was observed in C4 VSMCs, reaching nominal significance (p = 0.022, p.adj = 0.098) (Fig. 5A). Among all mRNA modification–related genes (median log_2_ normalized counts > 5; cf. Table S19), significant differences between C3 and C4 VSMCs were detected only for *TET1* (hm5C oxidase) after adjustment for multiple testing (p.adj< 0.05). No prominent global expression changes that could explain the observed RNA modification differences were detected (Fig. 5B), although any changes to mRNA-modifying enzymes may not have been detectable after the long incubation period.

**Fig. 5:**
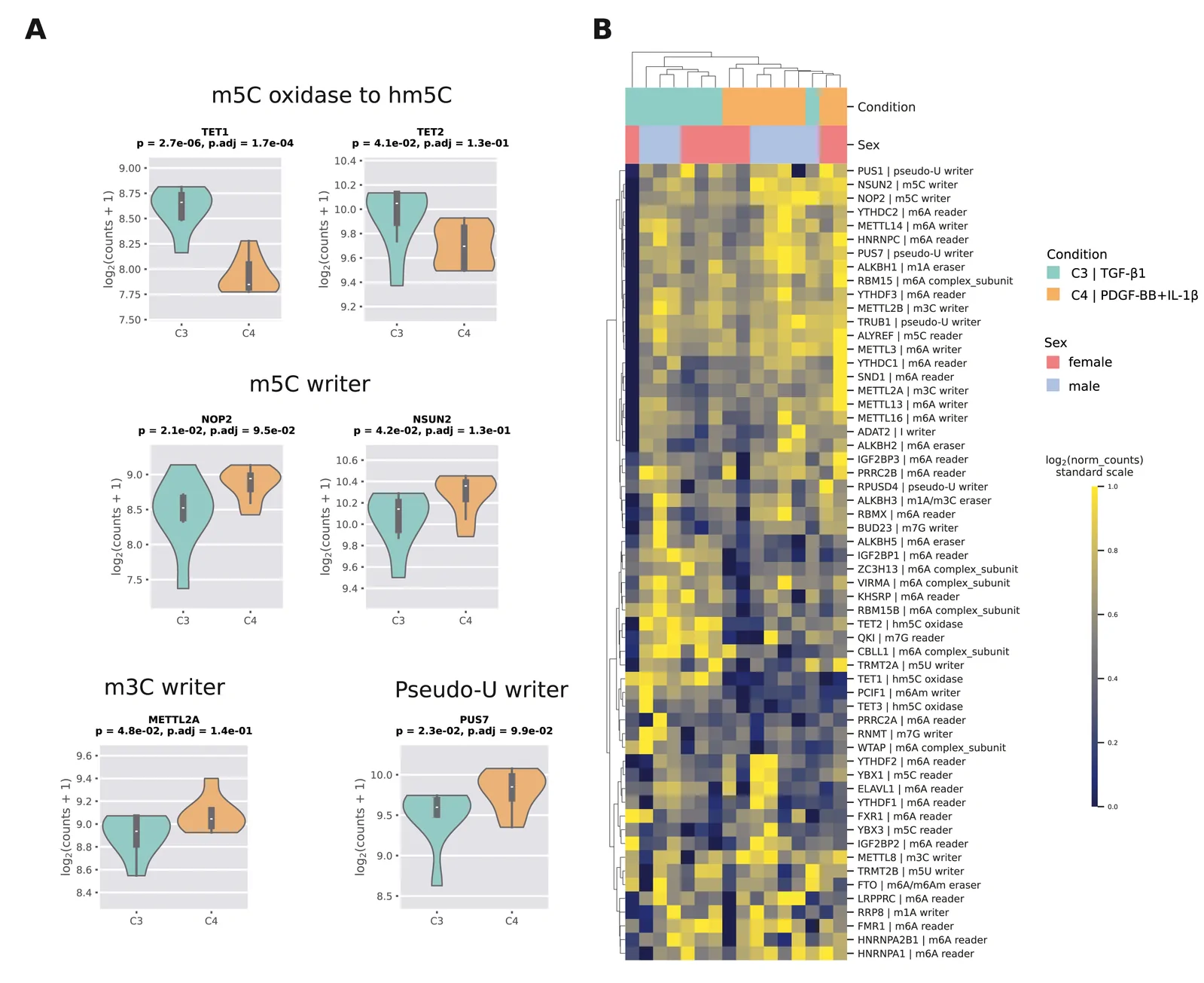
Expression pattern of genes encoding mRNA-modifying enzymes in C3 and C4 VSMCs (n = 8 biological replicates, i.e., cell lines, per condition). (**A**) Violin plots of genes whose canonical transcripts are differentially expressed in C3 vs. C4 at nominal significance (p < 0.05; for TET1: p.adj < 0.05; Table S6). (**B**) The clustermap shows the standardized expression level of genes with median log_2_ (normalized counts) ≥ 5 (Table S19).

### 3.5 RNA base modifications are associated with poly(A) tail dynamics

Next, we examined how DMR sites are associated with mRNA processing and translational regulation, including poly(A) tail dynamics. Density plots of poly(A) tail lengths in TGF-β1–treated VSMCs (C3) and PDGF-BB/IL-1β–treated VSMCs (C4) reveal that genes/transcripts with an increasing number of DMR sites exhibit progressively longer poly(A) tails, along with more pronounced differences between the two VSMC phenotypes (Fig. 6A). In addition, we observed a moderate correlation between gene/protein log_2_ fold changes and corresponding nominally significant shifts in poly(A) tail length (p ≤ 0.05). Longer poly(A) tails appear to be associated with lower mRNA levels as well as protein levels in C4 VSMCs (Fig. 6B). This correlation was weaker in genes without detectable DMRs and was no longer apparent when all transcripts were included, but remained evident in genes associated with at least three DMR sites (Fig. S12). This relationship is highlighted for *NAMPT* and *FN1* in Fig. 6C, which were among ten genes with significant differences in poly(A) tail length after adjusting for multiple testing (cf. Table S20). While *NAMPT* showed higher expression in C4 VSMCs, accompanied by shorter poly(A) tails, *FN1* exhibited the opposite pattern when all isoforms were considered. Interestingly, several uridines showed increased modification rates (in “higher” xPore mode) in C4 VSMCs. These modified uridines were located predominantly in the 3′-UTRs of *NAMPT* isoforms, whereas in *FN1* isoforms they occurred exclusively within coding sequences.

**Fig. 6:**
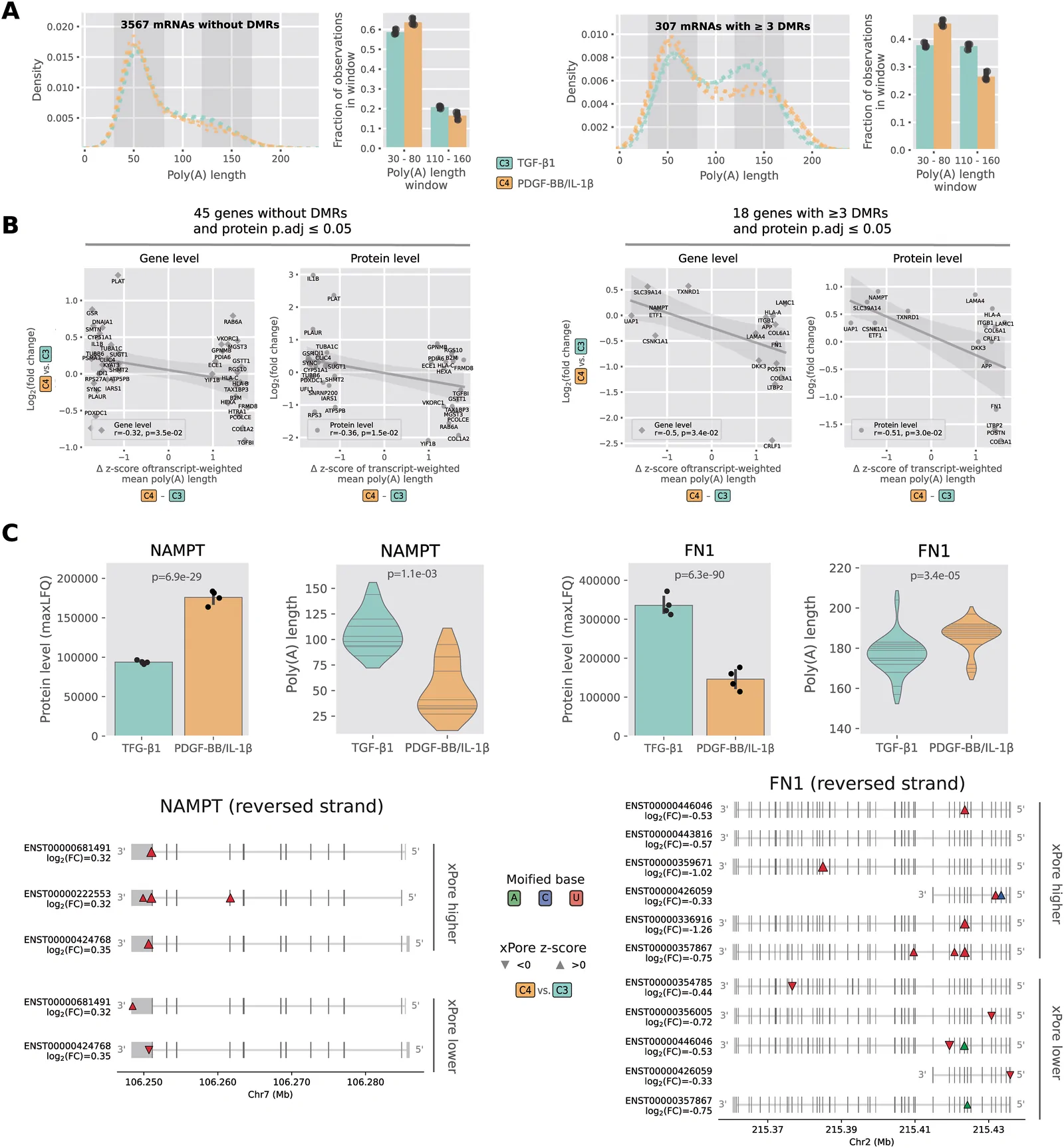
Exploratory analysis of poly(A) tail length distribution of mRNAs isolated from TGF-β1-stimulated (C3) or PDGF-BB/IL-1β–stimulated VSMCs (C4) (n = 3 technical replicates and n = 1 biological replicate). (**A**) Kernel density plots show increasing poly(A) tail length and greater differences between C3 and C4 with an increasing number of differential modification rate (DMR) sites. Dotted lines in the kernel density plots and the fraction of observations in the short-tail (30 - 80 nt) and long-tail (110 - 160 nt) windows are shown to illustrate the variation among the three replicates. (**B**) Pearson correlation plots of nominally significant differences (Δ) in gene-level z-score-normalized poly(A) tail length (n = 3 technical replicates and n = 1 biological replicate; transcripts per gene were aggregated, p ≤ 0.05) versus log_2_ fold change for differentially abundant proteins (p.adj ≤ 0.05) (n = 3 biological replicates for gene expression/n = 4 technical replicates for protein expression). Significance was assessed using a *t*-test. (**C**) Examples illustrating the relationship between protein abundance (n = 4 technical replicates and n = 1 biological replicate), poly(A) tail length, and DMR position (n = 3 technical replicates and n = 1 biological replicate). Poly(A) tail lengths across all isoforms (polya coverage > 10; FN1 = 9 isoforms, NAMPT = 3) and three technical replicates are shown per gene; p-values for protein levels are from proteomics analysis; significance of poly(A) length was assessed by Mann–Whitney U tests. Lower panels display isoforms with DMR sites stratified by xPore mode (“higher” or “lower”), with log_2_ fold change (C4 vs. C3) shown for each transcript.

### 3.6 Differentially modified uridine bases are enriched in predicted accessible RNA structural contexts in the 3′-UTR

Next, we predicted RNA secondary structures of 150-nucleotide sequences containing DMR sites from the comparison between C3 and C4 VSMCs using the ViennaRNA library to investigate whether differential modifications occur more frequently in distinct structural elements. In brief, 65 centroid RNA secondary structures were predicted per transcript and DMR position by shifting a 150-nucleotide window two bases downstream, starting from 140 nucleotides upstream of the DMR position. Centroid RNA secondary structures were clustered based on similarity scores. The RNA secondary structure with the lowest MFE of the most representative cluster (selected based on cluster size, median MFE, and MFE variance) was taken for over-representation analysis (Fig. 7A). As part of our quality control, we examined the negative minimum free energy (MFE) of all predicted centroid RNA secondary structures across relative cDNA positions. Consistent with previous observations, the negative median MFE per position declines toward the 3′-UTR, accompanied by reduced GC content, a hallmark of functional mRNAs [43] (Fig. 7B). Over-representation analysis of DMR sites in RNA secondary structure elements revealed that differentially modified uridines are 1.7-fold enriched in interior loops (*i.e.*, iiiii motif; p.adj = 0.025) in 3′-UTRs (Fig. 7C and Table S21).

**Fig. 7:**
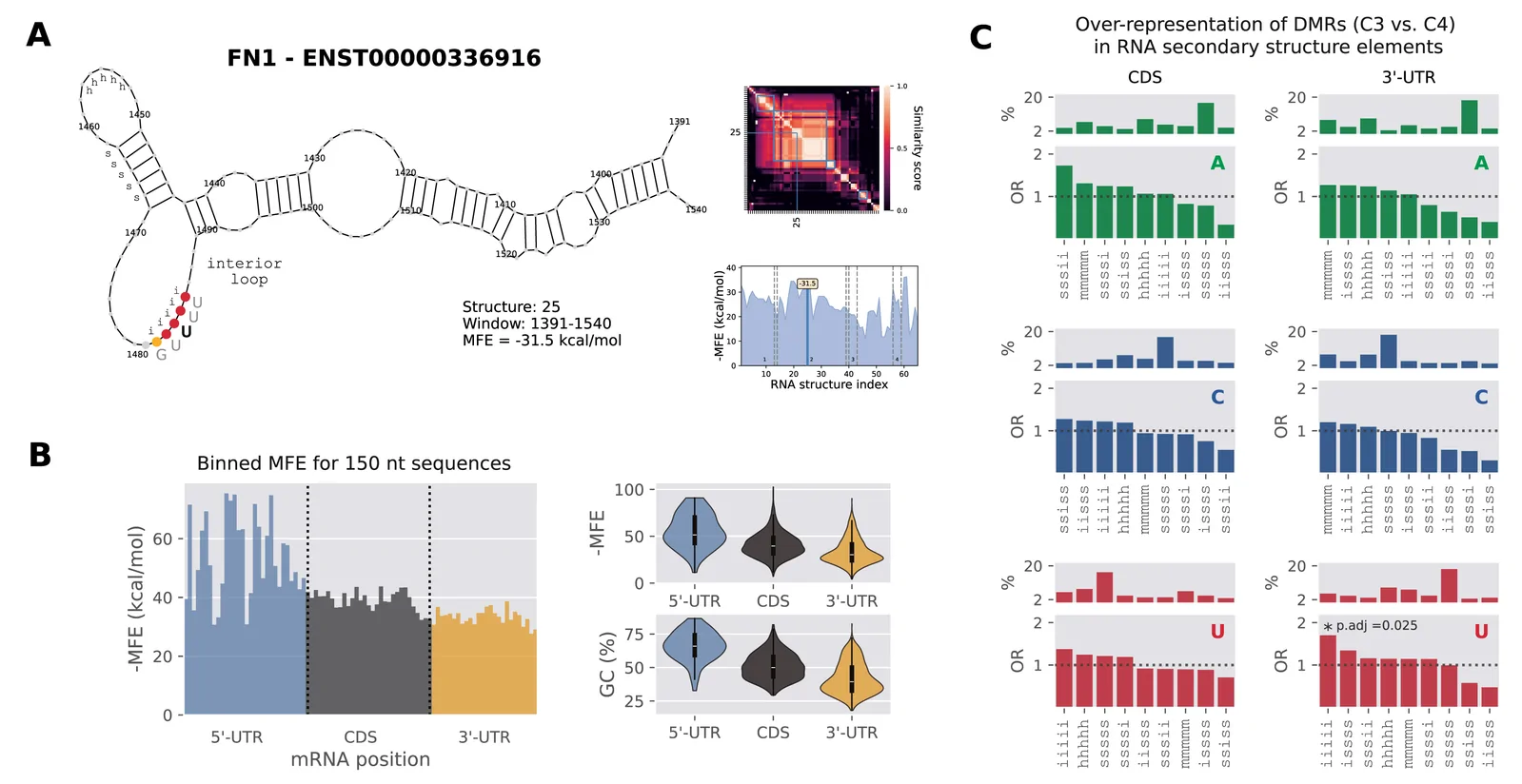
Distribution of differentially modified RNA bases from the comparison of TGF-β1 (C3) versus PDGF-BB/IL-1β (C4) in RNA secondary structures. (**A**) RNA secondary structures surrounding differential modification rate (DMR) sites were predicted from sliding 150-nt windows using the ViennaRNA package. Centroid structures with minimal free energy (MFE) were clustered, and dominant secondary structure elements (5-mers) were extracted. (**B**) Mean MFE across binned relative DMR positions (90 bins) in 5’/3′-UTR (untranslated region) and coding sequence (CDS). Violin plots highlight the correlation of MFE and GC content. (**C**) Differentially modified uridines are enriched in predicted interior loops (iiiii) within the 3′-UTR (Fisher's exact test). Bar plots show relative abundance (%) and odds ratios (OR) of secondary structure elements across 343 A, 571 C and 1146 U DMR sites (cf. Table S21). Here, relative abundance (%) denotes the proportion of DMR-centered structural 5-mers assigned to each secondary-structure element across all selected centroid structures. Secondary-structure elements were classified as stems (s), hairpin loops (h), multiloop segments (m), and interior loops (i). P values were Benjamini–Hochberg adjusted.

### 3.7 Modified RNA bases may modulate miRNA binding

We detected an excess of modifications near stop codons at the beginning of the 3′-UTR (cf. Fig. 2C), which has also been reported previously by the developers of xPore [8]. We hypothesize that this enrichment is related to DMR sites that influence miRNA binding, as miRNA-mRNA binding has been previously reported to be most effective when occurring at the beginning or the end of long 3′-UTRs [44]. Therefore, we assessed the potential impact of DMR sites on miRNA binding in C3 and C4 VSMCs. We quantified miRNA expression in four VSMC states (C2 to C5) and screened highly expressed miRNAs in conditions C3 or C4 (log_2_ (count) ≥ 10 in one replicate of C3 or C4) for miRNA-binding sites based on direct 8-mer seed matches. We identified 59 of the 195 highly expressed miRNAs whose seed sequences could potentially bind to DMR-containing mRNA regions in the 3′-UTR (Fig. 8A and Table S22, Table S23).

**Fig. 8:**
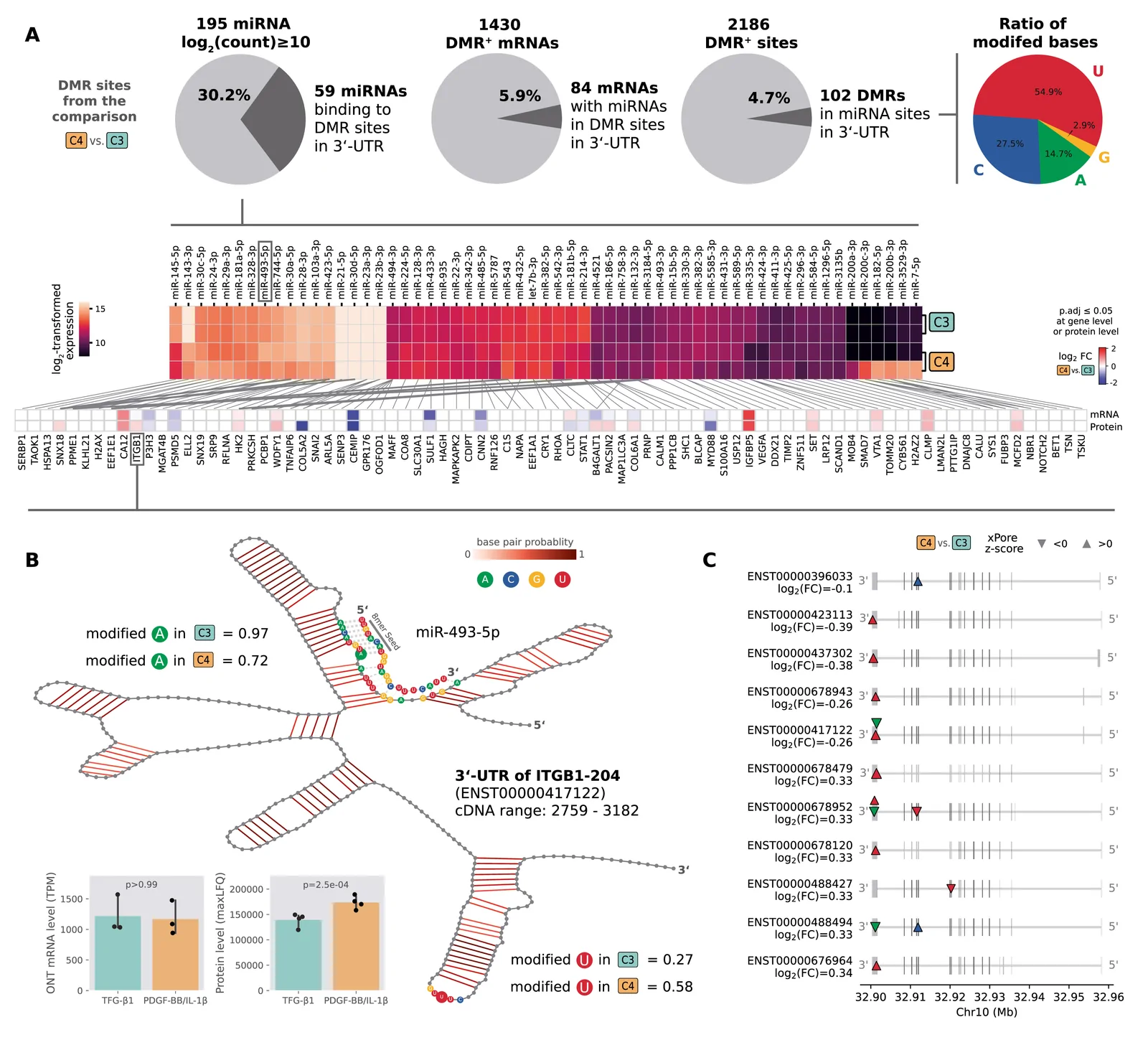
Exploratory analysis of potential miRNA binding at differential modification rate (DMR) sites for C4 vs. C3. (**A**) Pie charts summarize highly expressed miRNAs (log_2_ count ≥ 10; n = 2 technical replicates and n = 1 biological replicate) predicted to bind DMR sites via 8-mer seed sequences, the fraction of DMR site-positive transcripts and DMR sites within 3′-UTR miRNA binding sites, and the proportion of modified bases at these sites. Expression pattern of highly expressed miRNAs with at least one predicted DMR-binding site is shown across the conditions C3 and C4 (C3 = TGF-β1, C4=PDGF-BB/IL-1β; n = 2 technical replicates and n = 1 biological replicate). The log_2_ fold changes at gene level (from ONT RNA-Seq, n = 3 technical replicates and n = 1 biological replicate) and protein level (n = 4 technical replicates and n = 1 biological replicate) of predicted target genes are shown below. Genes/proteins showing no significant difference (p.adj > 0.05) between C3 and C4 at either the gene or protein level are displayed in white for clarity. (**B**) As an example, the *ITGB1* isoform ITGB1-204 illustrates a modified adenosine within a validated miR-493-5p binding site [45], showing reduced modification in PDGF-BB/IL-1β–treated cells accompanied by increased ITGB1 protein levels (n = 4 technical replicates and n = 1 biological replicate) at comparable expression levels (n = 3 technical replicates and n = 1 biological replicate). TPM: transcripts per million; maxLFQ: maximum Label-Free Quantification; p-value from proteomics analysis (see Supplemental Methods). A second DMR site was identified downstream, with differential uridine modification in C4. (**C**) Overview of DMR sites across ITGB1 isoforms, with log_2_ fold change (FC; C4 vs. C3) shown below each Ensembl transcript (n = 3 technical replicates and n = 1 biological replicate). Triangles indicate increased or decreased modification.

The highest number of predicted DMR binding sites across C3 and C4 was detected for miR-494-3p (n = 6), followed by miR-186-5p, miR-200b/c-3p, and miR-28-3p (n = 4) (Fig. 8A). DMR site-miRNA pairs were found for 84 mRNAs and 102 DMR sites, thereby affecting about 6% of all DMR site-positive transcripts. Of those, 56 sites have uridine modified, 28 cytidine, 15 adenosine, and 3 guanosine (Fig. 8A). However, only six predicted miRNA–target interactions had experimental support and a corresponding publication recorded in miRTarBase. In addition, the specific binding site of miR-493-5p (Fig. 8B) at the unique DMR-site AA-A-UG of *ITGB1-204* in the 3′-UTR (cDNA position 2859) was the only site that has been previously experimentally confirmed [45] but not yet recorded in miRTarBase. Fig. 8B illustrates a possible miRNA binding model postulating that miR-493-5p directly binds to a differentially modified adenosine located in the AA-A-UG sequence. Another DMR site (GU-U-UC) is located about 230 nucleotides downstream at the tip of a hairpin loop. Interestingly, the mRNA levels were similar in C3 and C4 VSMCs, yet integrin β1 protein levels appeared to be higher in the latter VSMC phenotype. Notably, the miRNA-binding sequence is conserved across all *ITGB1* isoforms, where modified uridines or adenosines lie nearby (Fig. 8C).

In order to investigate whether increased levels of miRNAs can influence RNA base modification, *e.g.*, by occupying the modifiable site, we screened miRNAs in phenotypic transition conditions C2 to C5 (Table S23 and S24) for expression changes between phenotypes. In total, 131 miRNAs were significantly differentially expressed across all four conditions, with the four most significant ones being miR-145-5p, miR-204-5p, miR-146a-5p, and miR-5585-3p. In the comparison C3 vs. C4, 41 significantly differentially expressed miRNAs (Wald test for significant fold change differences; p.adj < 0.05) were identified. Of these, miR-145-5p showed the greatest expression difference, exhibiting a ∼4-fold higher abundance in C3 VSMCs compared with C4 VSMCs (Table S24). This is consistent with prior studies demonstrating time- and dose-dependent downregulation of miR-145-5p under PDGF stimulation [46]. The miRNA miR-145-5p and ten additional differentially expressed miRNAs (C4 vs. C3) have predicted mRNA binding sites coinciding with DMR sites (Table S24). However, we did not find evidence that altered miRNA levels influence RNA base modifications. For example, miR-145-5p is predicted to bind at a DMR site (ACUGG) in a *SERBP1* transcript (see Fig. 8A) that exhibits a lower modification rate in C4 compared with C3 VSMCs (DMR = −0.33). In contrast, miR-143-3p, which is also expressed ∼4-fold higher in C3 VSMCs, is predicted to bind at DMR sites in *HSPA13* and *TAOK1* (both UCUCA), where the modification rate is higher in C4 VSMCs (DMR = 0.25 for *HSPA13* and DMR = 0.44 for *TAOK1*). Of note, neither of these predicted target genes showed differences at gene-level expression or protein abundance (Table S25).

## 4 Discussion

Using Nanopore direct RNA-Seq, we performed a transcriptome-wide, untargeted screening of RNA base modifications in vascular smooth muscle cells (VSMCs) treated with either TGF-β1 (C3) or PDGF-BB/IL-1β (C4). Our main findings suggest that (1) up to 5% of mRNA bases appear to be modified (*e.g.*, methylated); (2) Differences in mRNA base modifications between an intermediate contractile, ECM-producing C3 VSMC state and pro-inflammatory C4 VSMC phenotype may often occur near stop codons and at the beginning of 3′-UTRs, specifically affecting uridines in predicted accessible 3′-UTR regions; (3) The GY-U-YY (Y=U or C) appeared to be the most prominent motif found in ∼26% of isoforms; and (4) DMR sites might be linked to translational activity, *e.g.,* by regulating poly(A) tail dynamics and miRNA binding.

These exploratory findings pave the way toward understanding VSMC phenotype switching in atherosclerosis beyond established transcriptional and epigenetic regulatory mechanisms [4]. Although we were not able to detect traces of mRNA oxidation in oxidatively stressed VSMCs (C1) using Nanopore direct RNA-Seq, our multi-level analysis suggests that mRNAs can harbor multiple types of cytokine-induced base modifications. Further, nominal expression changes in *PUS7* and several cytidine-modification-associated genes (*TET1, TET2, NOP2,* and *NSUN2*) indicate potential regulators of the altered U- and C-centered nanopore signals in C4 VSMCs, but require independent validation. Future studies combining cytokine time courses with pathway or transcription-factor perturbation and orthogonal modification mapping will be required to define the regulation and kinetics of candidate mRNA-modifying enzymes and their target sites.

The most strongly enriched sequence context in C4 versus C3 VSMCs appeared to be the GY-U-YY motif (Y = pyrimidine base, C or U). To our knowledge, this motif has not been described so far and presents a potential target for future research. More broadly, genes associated with multiple DMR sites showed strong transcript–protein concordance and were enriched among differentially abundant proteins. These included the atherosclerosis-relevant genes *FN1*, *POSTN*, *COL3A1*, and *CALD1*, which have been previously linked to atheroprotective VSMC phenotypes and plaque stability [5,42]. Together, these observations suggest that differential RNA modifications may contribute to VSMC phenotypic regulation in atherosclerosis, although further mechanistic studies are required to establish causality.

To elucidate regulatory mechanisms underlying differential modification rate (DMR) sites, we investigated multiple possible consequences of DMR sites on mRNA processing. We analyzed the impact of DMR sites on mRNA splicing but did not find evidence for pathway-relevant differences in isoform usage in a DMR-dependent manner. In fact, DMR sites were often found in proximity to stop codons in agreement with previous studies [8,47], instead indicating regulatory activity that might impact mRNA usage, mRNA folding, mRNA stability, or miRNA binding, and thus translational output.

First, we observed an apparent negative correlation between protein abundance and poly(A) tail lengths, particularly for transcripts harboring DMR sites, which is in line with a previous study that also applied Nanopore direct RNA-Seq [47]. Indeed, the potential contribution of DMR sites highlights the regulatory potential of these modifications.

Second, uridine-centered modifications appeared to be enriched in predicted single-stranded RNA structure elements in 3′-UTRs, supporting the regulatory nature of these mRNA modifications, as these regions are potentially accessible for RNA-modifying enzymes and allow binding of proteins that exert post-transcriptional regulatory effects. The vast majority of significant differential modifications affected uridine, although the specific identities of these modifications remain unresolved.

Third, it has been demonstrated that mRNA modifications can potentially modulate miRNA binding when located in seed match regions in 3′-UTR miRNA binding sites [48]. Notably, 59 out of 195 highly expressed miRNAs (in C3 or C4) could directly bind to DMR sites with their seed sequence. This exploratory screen provides candidate DMR-affected miRNA-target interactions that can be evaluated in future studies for prediction confidence and prioritized for functional validation. Because miRNA profiling was performed primarily to identify highly expressed miRNAs and not to rigorously quantify differential miRNA expression across phenotypes, confidence in reported phenotype-specific miRNA expression changes is limited, and a potential link with DMRs should be considered exploratory and hypothesis-generating only.

While this study is the first to comprehensively integrate short-read sequencing, long-read direct RNA sequencing, and mass spectrometry, such multi-platform integration has technical limitations. For example, there is still a discrepancy between short-read RNA-Seq and long-read RNA-Seq with respect to determining expression estimates [49], as well as mapping of reads. Further, quantification of long reads can be affected by incomplete transcript references, and recently, a large number of novel transcripts have been detected in an experimental setup similar to ours [19]. Despite the higher mapping accuracy of long-read sequencing, the limited throughput of MinION flow cells restricted ONT analyses to highly expressed genes that exhibit similar expression levels in two conditions, preventing differential modification testing for many transcripts. Consequently, insufficient coverage and limited replicates likely led to a considerable proportion of missed DMR sites, especially in medium- and lowly expressed transcripts. This technical limitation may explain the observation of more DMR sites in genes with high protein abundance. It may also underlie the low number of detected modifications in C1 VSMCs despite differential expression of multiple genes related to mRNA modification. Moreover, the time point of DMR assessment after cytokine stimulation might be crucial, as some RNA base modifications might lead to rapid mRNA degradation.

A further challenge in assessing the regulatory impact of DMR sites on translational output is that the protein quantification relies on peptide-based measurements, which generally permit assignment at the gene level but not at the transcript level, thereby limiting the linkage of isoform-resolved DMR sites to protein expression.

Another limitation is the use of the modification-type-agnostic detection approach. Although k-mer context and enzyme-related gene expression can suggest specific modification types (*e.g.*, the DRACH motif for m6A), we did not detect strong signals of known sequence motifs at differential sites. Moreover, the ONT RNA002 chemistry precluded the use of ONT basecalling models for specific modifications, which are currently available only for RNA004 chemistry [50] (cf. https://genesilico.pl/modomics/tools). Consequently, accurate classification of individual, specific modification types will require dedicated technologies and experimental validation in future studies.

In conclusion, our modification-type-agnostic approach provides a comprehensive, transcriptome-wide view of the RNA modification landscape underlying VSMC phenotypic transitions. We show that distinct RNA modification types frequently co-localize within the same transcripts, particularly near stop codons, suggesting combinatorial regulatory effects on processes such as polyadenylation, mRNA stability, and interactions with regulatory proteins or miRNAs. Moreover, our data show that uridine-centered nanopore signals are associated with post-transcriptional regulatory features and nominate candidate mechanisms that may help maintain atherosclerosis-relevant VSMC phenotypes for future validation. Finally, modification sites may serve as targets for miRNA-based therapeutic strategies, and base modifications may be detectable in circulating cell-free RNA, highlighting their potential as biomarkers of disease severity.

## Resource availability

### Lead contact

Requests for further information and resources should be directed to and will be fulfilled by the lead contact, Inken Wohlers (iwohlers@fz-borstel.de).

### Materials availability

This study did not generate new unique reagents.

### Data and code availability

Key result files, scripts, and figures have been made publicly available at FAIRDOMHub under “OMICS of vascular smooth muscle cells under oxidative stress” and can be accessed at https://fairdomhub.org/projects/461. Transcriptome sequencing data (Illumina mRNA and small RNA-Seq as well as ONT direct RNA-Seq) for this study have been deposited in the European Nucleotide Archive (ENA) at EMBL-EBI under study accession number PRJEB98900 (https://www.ebi.ac.uk/ena/browser/view/PRJEB98900). Proteomics raw data files are available in PRIDE under accession ID PXD065513. The modification workflow is available at https://github.com/iwohlers/2025_ont_rnaseq_vsmc, and scripts used for integrative analyses and figure generation at https://github.com/ToReinberger/Epitranscriptomic_profiling_of_VSMC. A detailed method description, Supplementary Figures, and Supplementary Tables are available in the Supplemental Material.

## Author contributions

Conceptualization, T.R., I.W.; Data curation, T.R., A.I., A.W., E.H., I.W.; Formal analysis: T.R., A.I., A.W., E.H., I.W.; Funding acquisition: T.R., I.W.; Investigation: T.R., A.I., T.F., J.F., A.W., E.H., T.Z., I.W.; Methodology: T.R., I.W.; Project administration: T.R., I.W.; Resources: T.R., T.Z., I.W.; Software: T.R., A.I., I.W.; Supervision: T.R., I.W.; Validation: T.R., I.W.; Visualization: T.R., A.I., I.W.; Writing – original draft: T.R., I.W.; Writing – review & editing: T.R., A.I., T.F., J.F., A.W., E.H., T.Z., I.W.

## Declaration of generative AI and AI-assisted technologies in the manuscript preparation process

During the preparation of this work, the authors used Grammarly, Copilot, and ChatGPT for language refinement. After using these tools, the authors reviewed and edited the output as needed and take full responsibility for the content of the published article.

## Declaration of competing interest

The authors declare no competing interests.

## Data Availability

FAIRDOMHub: project 461; GitHub: ToReinberger/Epitranscriptomic_profiling_of_VSMC, iwohlers/2025_ont_rnaseq_vsmc; ENA: PRJEB98900; PRIDE: PXD065513
