# Supplementary material for: Epitranscriptomic profiling of VSMC phenotypes reveals uridine modifications linked to post-transcriptional regulation
Source: Biochem Biophys Rep. 2026 Sep 28;48:102816. doi: 10.1016/j.bbrep.2026.102816 (PMC13640025; doi:10.1016/j.bbrep.2026.102816)
Supplement: Supplementary file 2 [file mmc3.pdf]

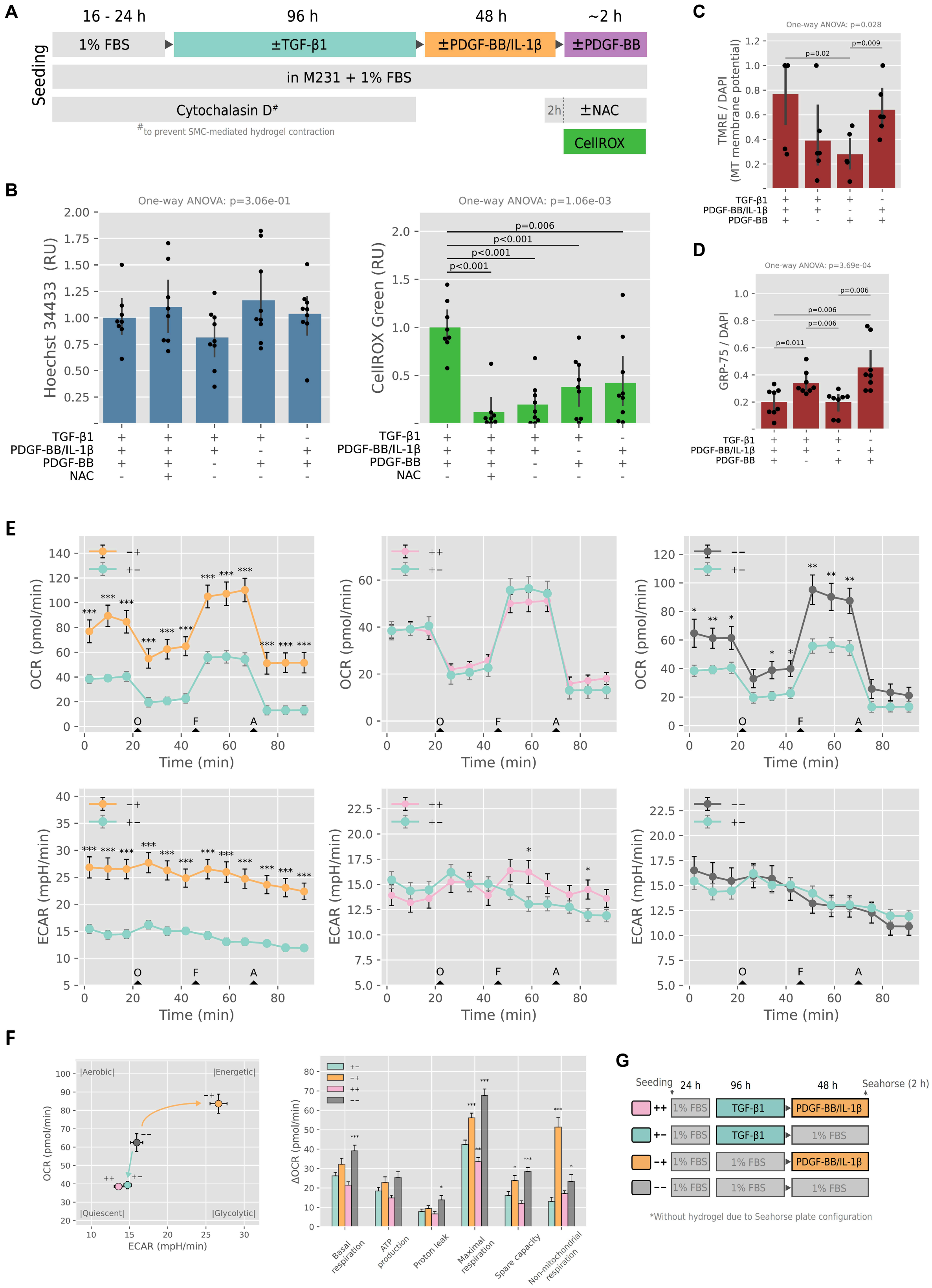

Figure S1: Oxidative stress and mitochondrial activity in vascular smooth muscle cells (SMCs) under TGF-β1 (condition C3) and PDGF-BB / IL-1β conditions. (**A**) VSMC culture and treatment scheme (FBS, fetal bovine serum; NAC, N-acetylcysteine). (**B**) Endogenous oxidative stress assessed by CellROX fluorescence, normalized to Hoechst 33342, following treatment with TGF-β1 (5 ng/mL), PDGF-BB (10 ng/mL) / IL-1β (10 ng/mL), or high-dose PDGF-BB (200 ng/mL). Two VSMC lines with

n=4 (HAOSMC IV) or n=5 (HAOSMC 1596) technical replicates were used. NAC (1 mM) was used to reduce oxidative stress. RU: relative unit. (**C**) Mitochondrial (MT) membrane potential measured by TMRE fluorescence; (**D**) mitochondrial abundance assessed by GRP75 immunostaining. (**E**) Oxygen consumption rate (OCR) and extracellular acidification rate (ECAR) measured by Seahorse analysis in VSMCs treated with TGF-β1 (+−), PDGF-BB / IL-1β (−+), both growth factors (++), or untreated (−−). Approximately 1 hour before measurement, the cell culture medium was replaced by Seahorse XF DMEM assay (Agilent #103681-100) supplemented with 1 mM Pyruvate (Gibco), 4 mM L-glutamate (Gibco), and 10 mM Glucose (Sigma). To measure changes in oxygen consumption rate (OCR) and extracellular acidification rate (ECAR) using a Seahorse XF24 analyzer, 1.4 µM oligomycin (O), 1 µM FCCP (F), and 1.5 µM antimycin A/1.5 µM rotenone (A) were added sequentially in each well. (**F**) Seahorse parameters were calculated according to the manufacturer's instructions. Each replicate value is the mean of three to five wells of Seahorse plates. (**G**) Experimental protocol for each condition. In **C** - **F**, one VSMC line with n=6 (TMRE), n=7 (GRP-75), and n=5 (Seahorse) technical replicates was used (cf. **Table S1**). In **B** - **F**, statistical analysis was performed by one-way ANOVA followed by unpaired Student's t-tests where applicable ($p < 0.05$). In **F**, statistical significance was calculated using a two-sided Student's t-test per time point.

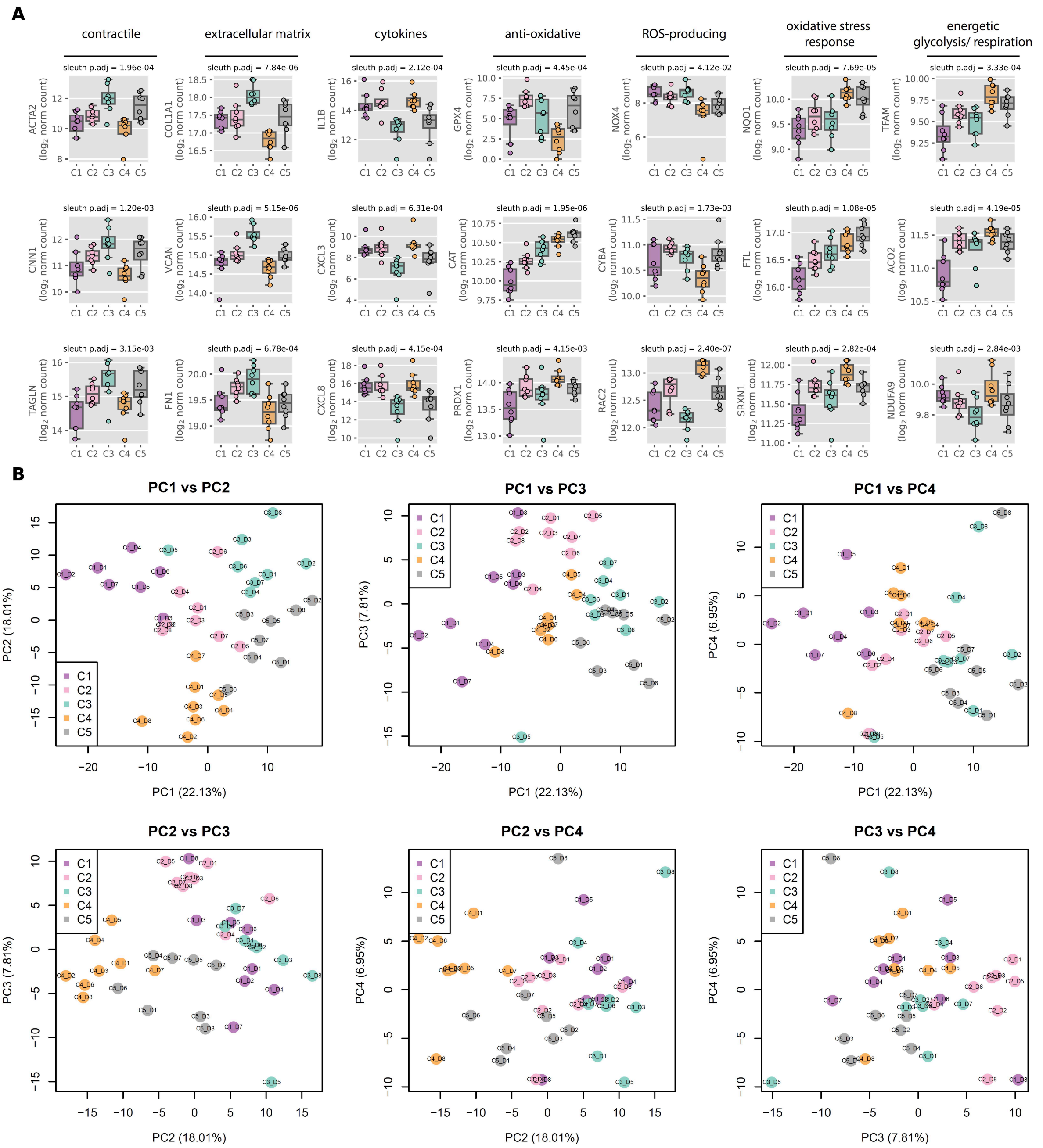

Figure S2*:* (**A**) Expression level of representative marker genes indicating different VSMC phenotypes. (**B**) Principal component-based transcriptome profiling of VSMC phenotypic conditions C1 to C5 based on Illumina short-read RNA sequencing of eight VSMC cell lines as biological replicates. Shown are all combinations of the principal components (PC) 1 to 4 for the 1,000 most variable transcripts after keeping the on average most highly expressed transcript per gene if its normalized and batch-corrected log2 expression is larger than or equal to 5 in all samples.

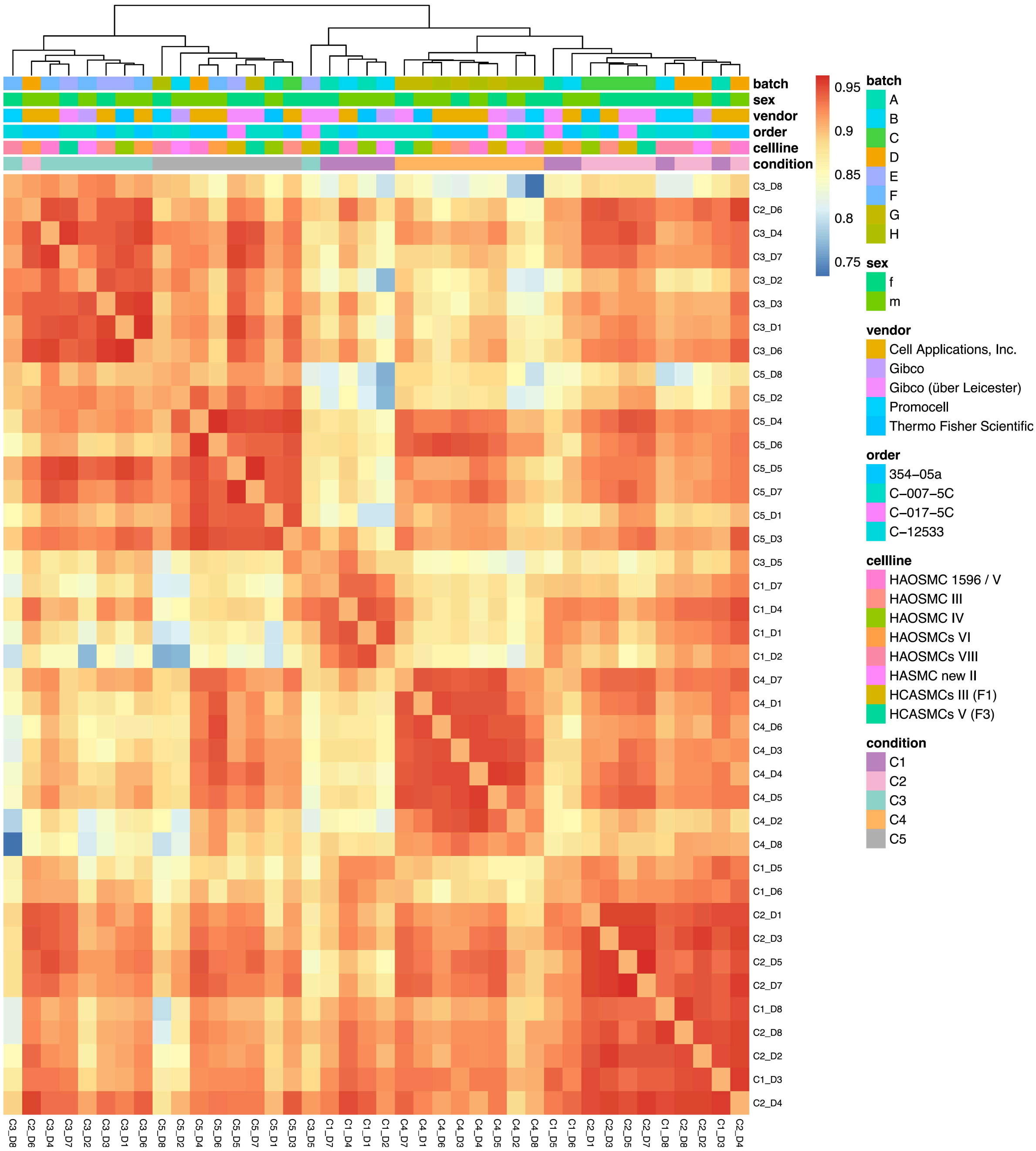

Figure S3*:* Sample-to-sample correlation heatmap based on pairwise Pearson correlations calculated from the 1,000 most variably expressed genes according to short read-based, log2-transformed expression after normalization across samples (n=8 biological replicates per condition). The hierarchical clustering uses 1–correlation as the distance. Typically, expression profiles of samples from the same condition are most strongly correlated. Expression profiles of oxidatively stressed samples (C1) tend to be less correlated with the profiles of samples of other conditions.

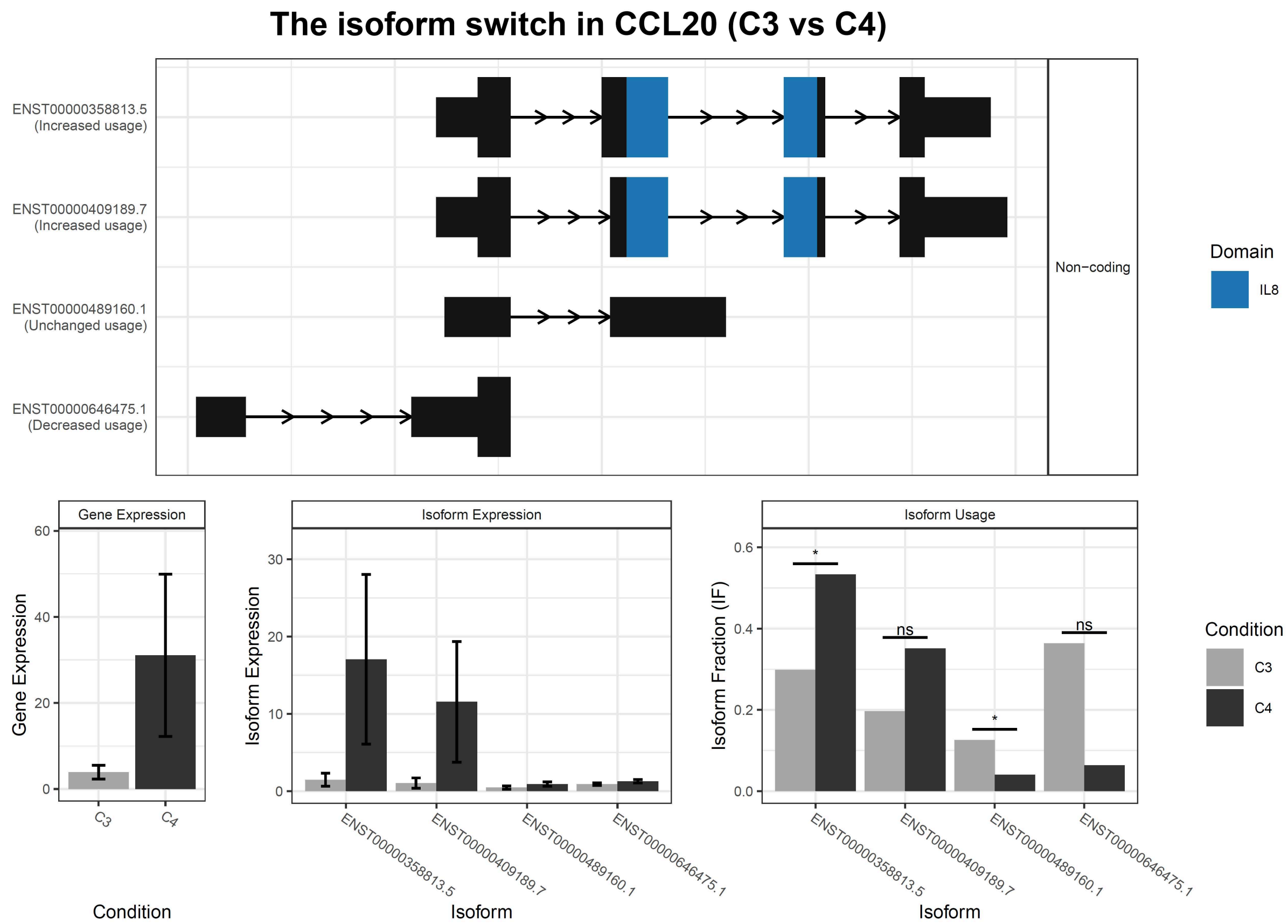

Figure S4: Isoform-usage analysis of *CCL20* from Illumina RNA-Seq data derived from vascular smooth muscle cells treated with TGF-β1 (C3) or PDGF-BB / IL-1β (C4) (n=8 biological replicates). Four of the five overall isoforms of *CCL20* are shown in A. Transcripts ENST00000358813.5, ENST00000409189.7, and ENST00000646475.1 are annotated as protein-coding in Ensembl, although predicted non-coding by the coding potential assessment tool (CPAT). The first two include an IL8 domain, shown in blue. Both show much higher expression than other isoforms in C4. Transcript ENST00000489160.1 is annotated as a retained intron. Significance is computed within the tool SwitchAnalyzeR. The top panel defines isoform usage (Increased/Unchanged/Decreased) based on an absolute difference of more than 10%, irrespective of significance. The lower right panel provides the statistical significance of the respective isoform usage difference. *, p-value<=0.05; **, p-value<=0.01; *** p-value<=0.001.

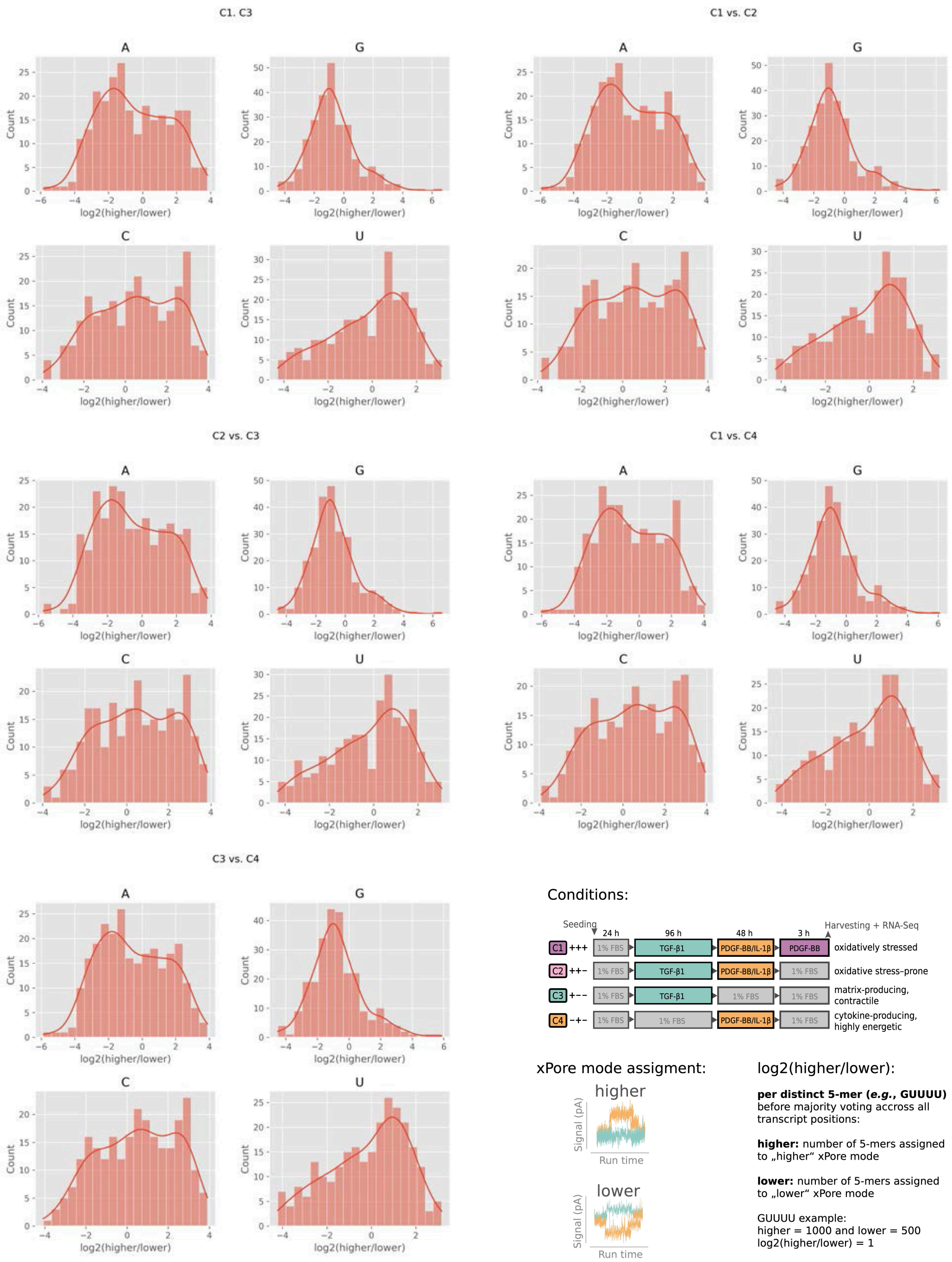

Figure S5: Histograms of xPore modification modes ("higher" vs. "lower") across conditions C1 to C4 for each RNA base (A, G, C, and U) before filtering xPore data (*i.e.,* majority voting) (n=3 technical replicates). The distribution pattern may reflect different base modifications. For example, modifications in G tend to result in lower raw Nanopore signals, while in A or C, at least two distinct base modifications can be suspected.

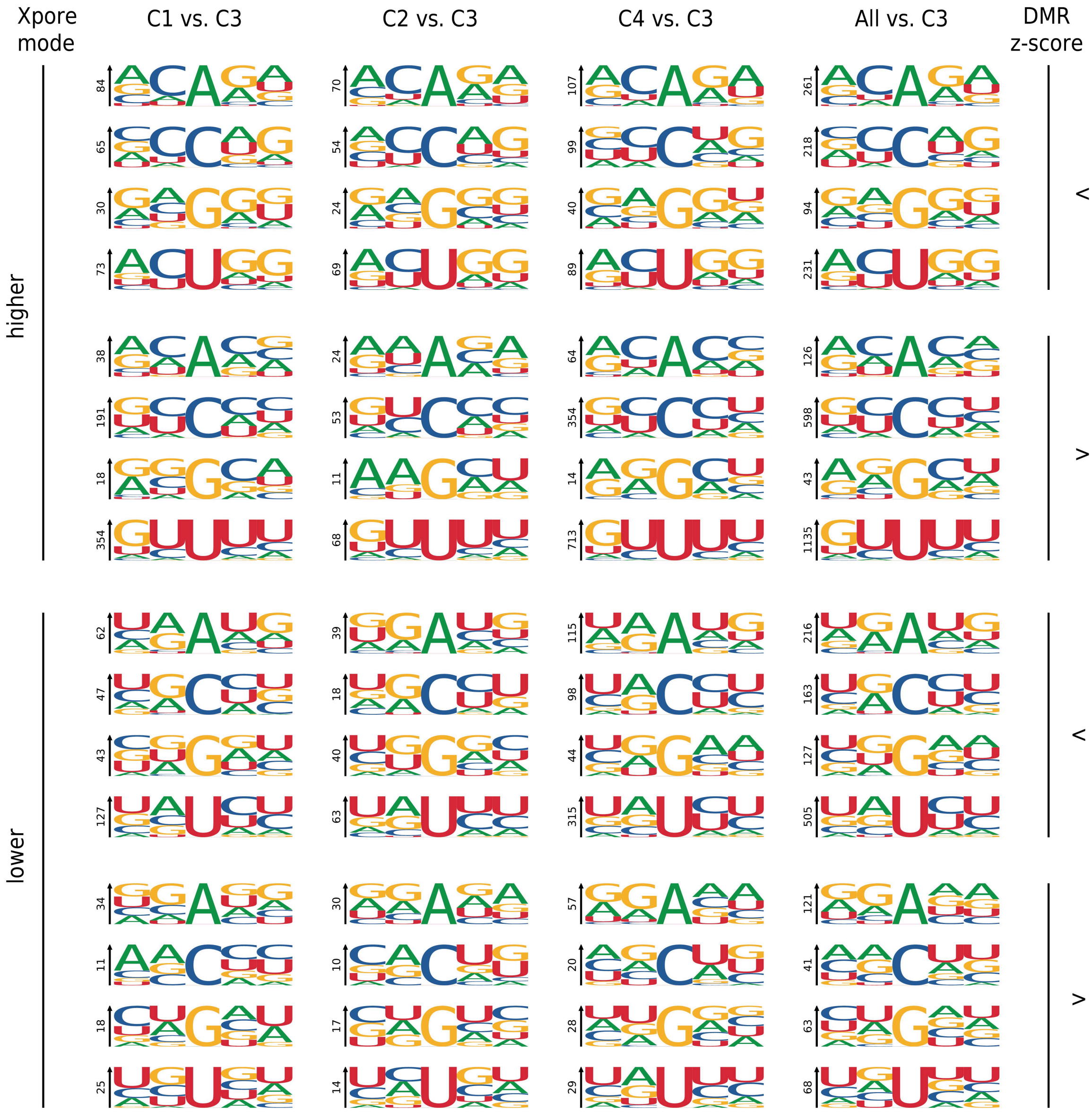

Figure S6: Number of significant sites as 5-mer motifs with differential modification rates more than 25% in comparisons with TGF-β1-treated condition C3 (n=3 technical replicates per condition). The sites are stratified by xPore mode ("higher" or "lower") and direction of modification change (reflected by a z-score less than or greater than 0). Each 5-mer motif is generated across the sites with the respective mode and direction, with the number of significant sites denoted. Most prevalent are uracil modifications, and most prominent is the motif GUUUU across all comparisons (C1, C2, and C4; PDGF-BB / IL-1β-treated smooth muscle cells) with C3.

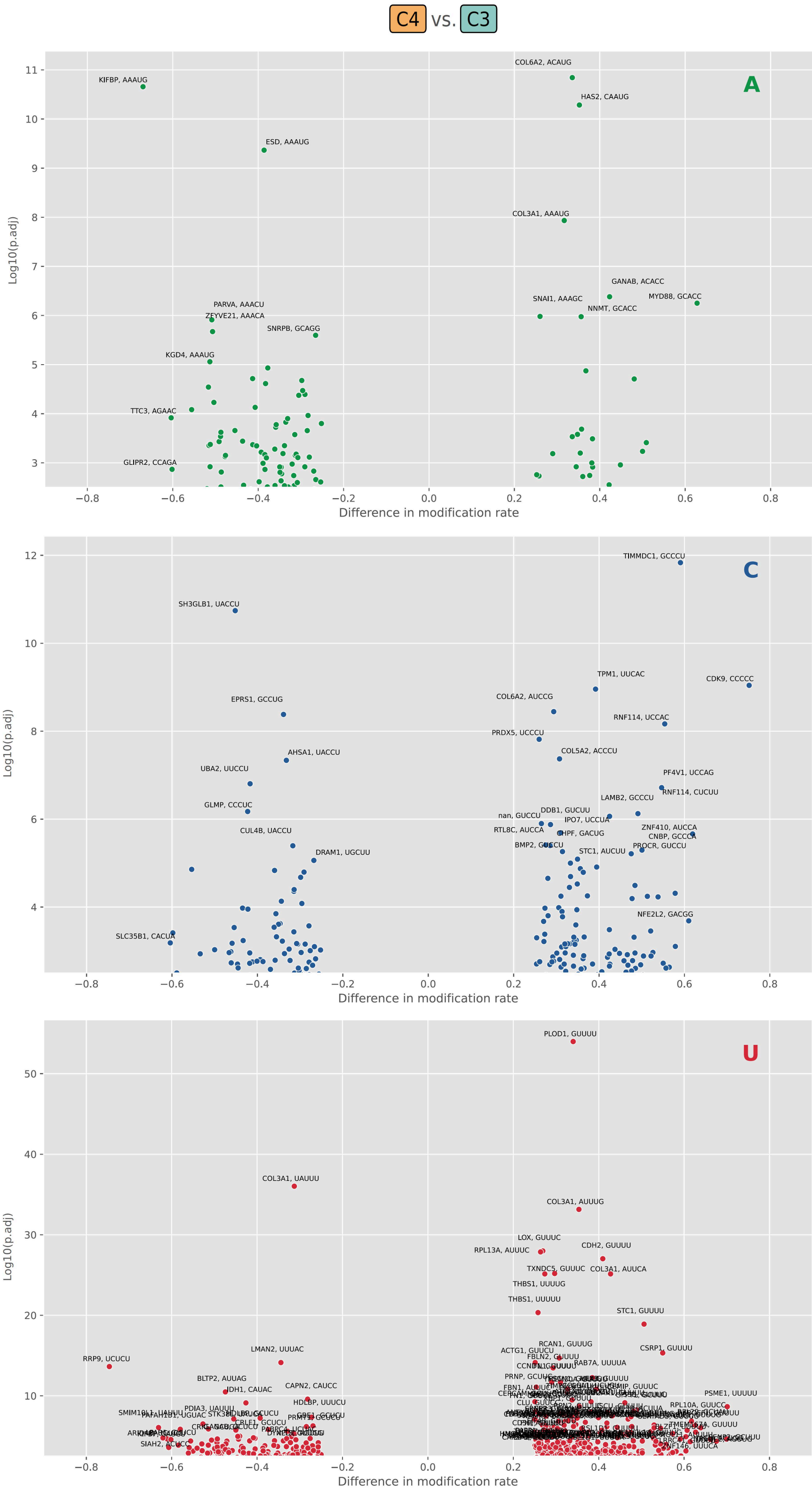

Figure S7: Volcano plots of differentially modified RNA bases in vascular smooth muscles treated with TGF-β1 (C3) or PDGF-BB / IL-1β (C4) (n=3 technical replicates per condition).

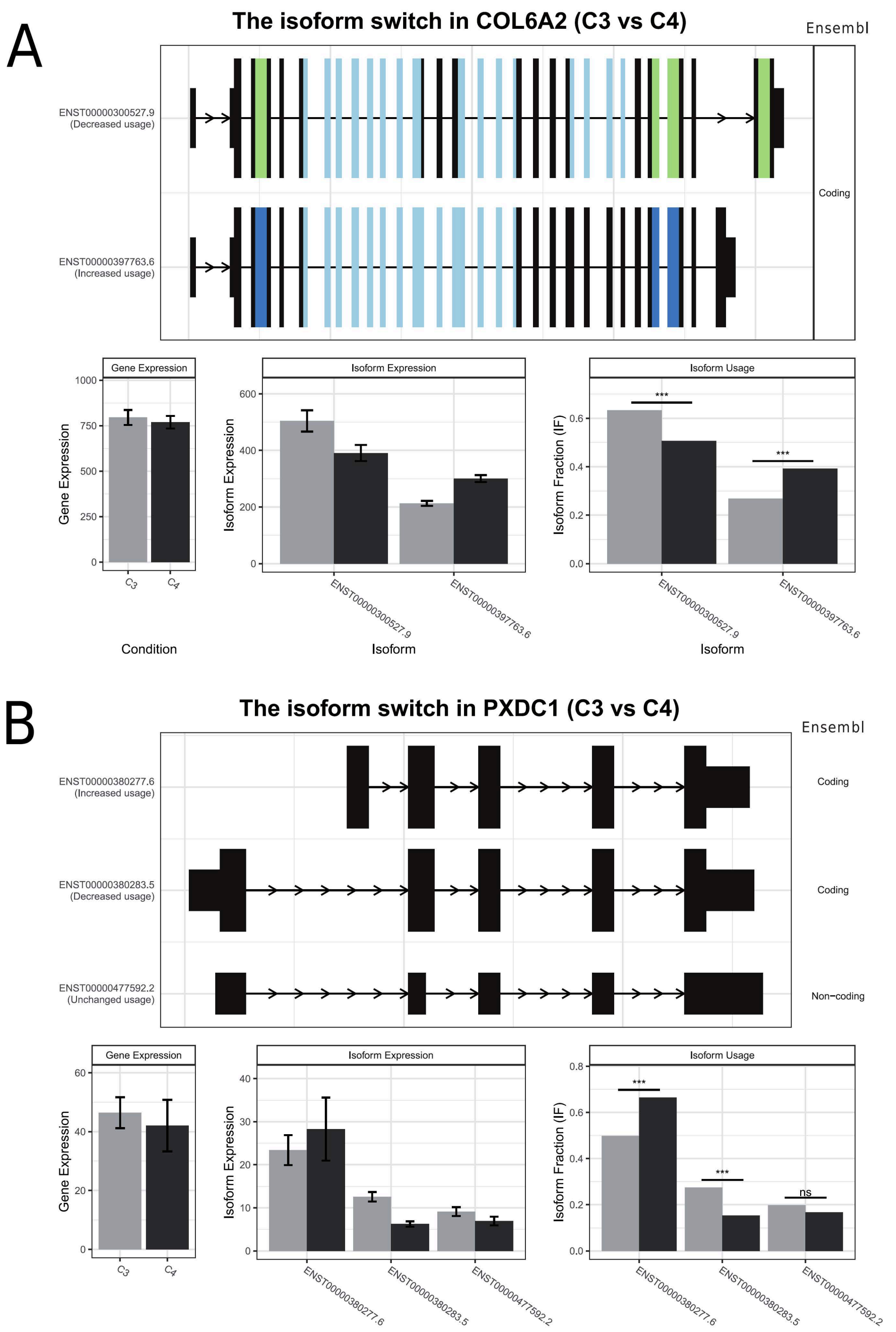

Figure S8: Isoform-usage analysis of two DMR-affected genes with significant differences in isoform usage (**A**) *COL6A2*, and (**B**) *PXDC1* from Illumina RNA-Seq data derived from vascular smooth muscle cells treated with TGF-β1 (C3) or PDGF-BB / IL-1β (C4) (n=8 biological replicates). Significance is computed within the tool SwitchAnalyzeR. The top panel defines isoform usage (Increased/Unchanged/Decreased) based on an absolute difference of more than 10%, irrespective of significance. The lower right panel provides the statistical significance of the respective isoform usage difference. *, p-value<=0.05; **, p-value<=0.01; *** p-value<=0.001.

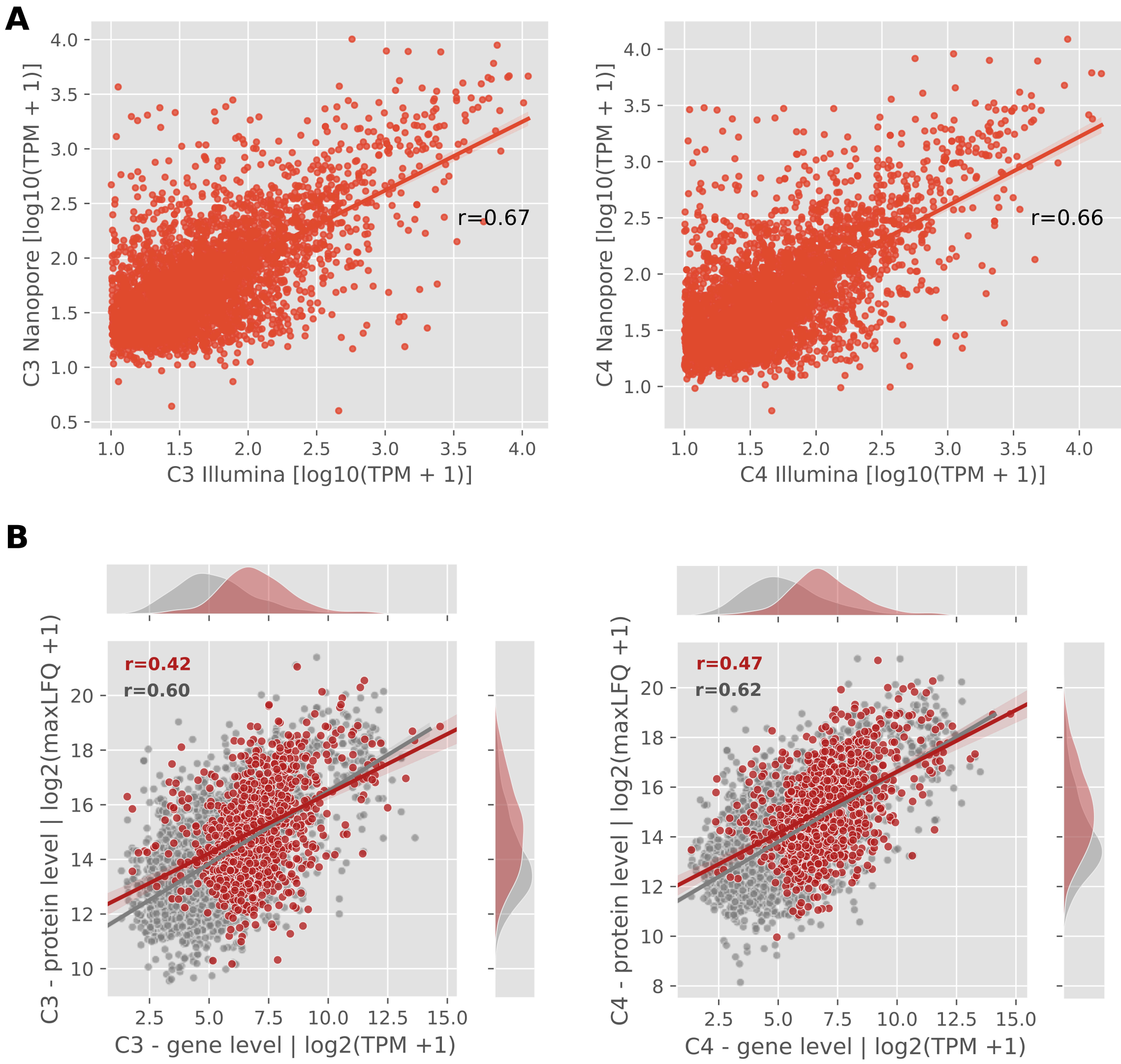

Figure S9: (**A**) Correlation between Nanopore (n=3 technical replicates) and Illumina (n=8 biological replicates) log10-transformed mRNA levels with >10 transcripts per million (TPM). Expression levels are means of three technical replicates (Nanopore) or levels derived from one Illumina sequencing run with the same VSMC line used for Nanopore sequencing (HAOSMC IV; cf. **Table S1**). The Pearson's correlation coefficient (r) is shown for VSMCs treated with TGF-β1 (left panel; C3; 3,843 transcripts) or PDGF-BB / IL-1β (right panel; C4; 3,808 transcripts). (**B**) Regression analysis of gene-level versus protein-level expression in C3 and C4 for genes and corresponding transcripts with (red) or without (gray) differentially modified RNA bases (DMR sites; C3 vs. C4). The p-value was <0.001 for all analyses and calculated with a two-sided t-test as implemented in the SciPy stats package.

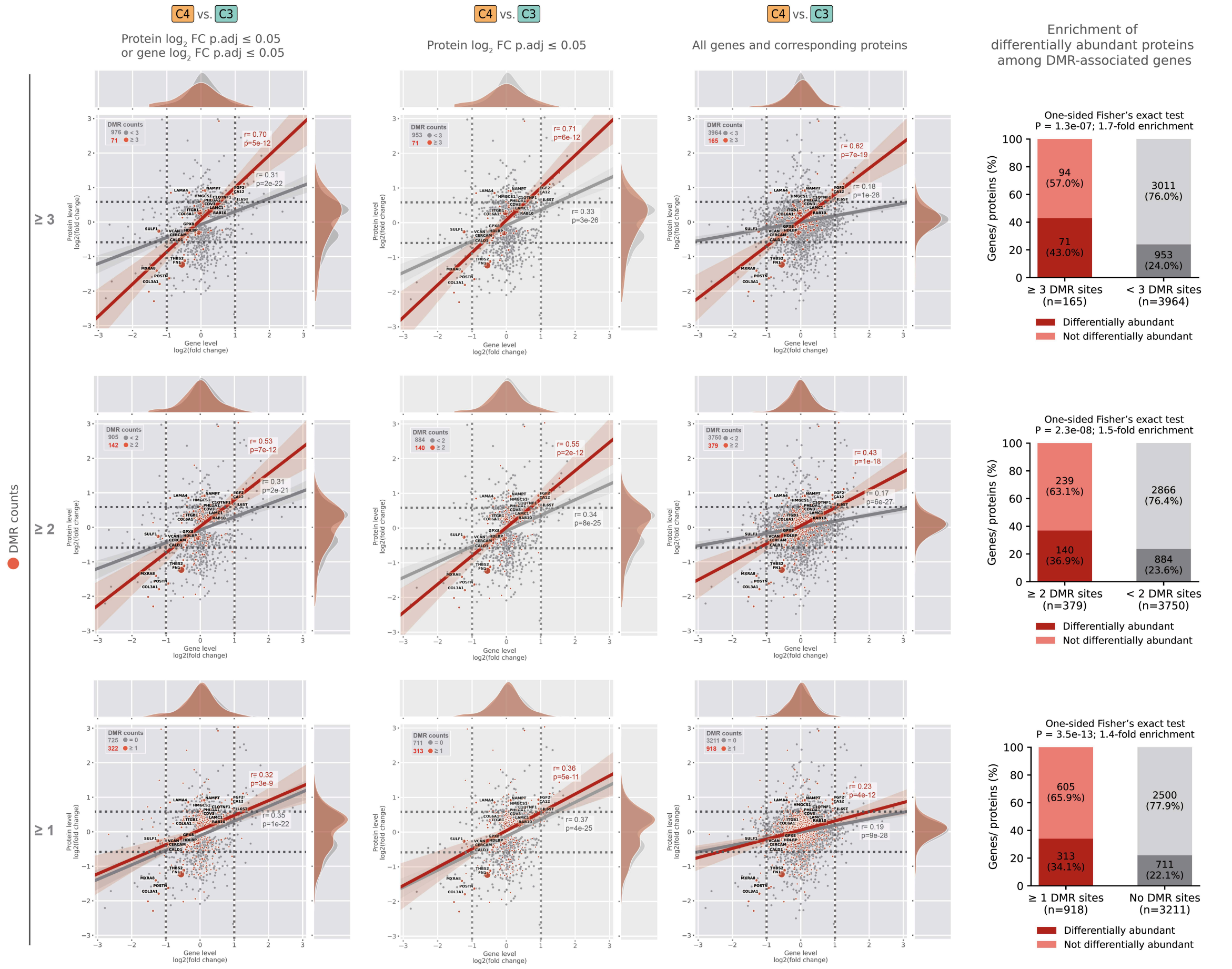

Figure S10: Association between differential modification rate (DMR) site burden and gene/protein abundance in vascular smooth muscle cells. Differential proteomic analysis of VSMCs treated with TGF-β1 (C3) or PDGF-BB / IL-1β (C4) (n = 4 technical replicates per condition). The Pearson correlation (r; significance assessed with a t-test) was calculated for genes with varying DMR sites across all isoforms. The stacked bar plots on the right show an enrichment of differentially abundant proteins among DMR-associated genes/proteins. Significance was assessed using a one-sided Fisher's exact test.

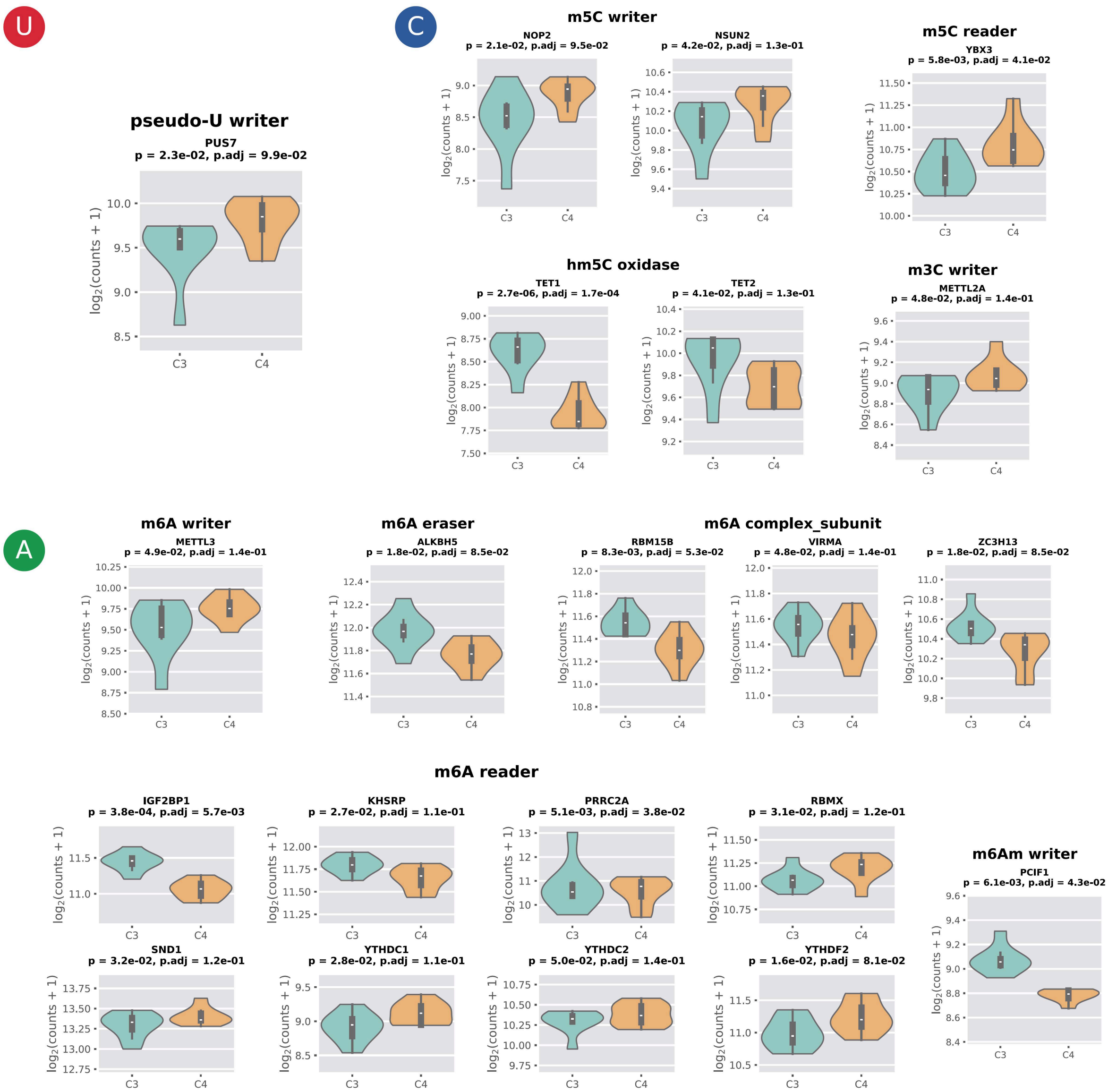

Figure S11: Gene expression of differentially expressed genes (nominally significant; $p < 0.05$) encoding proteins that might alter RNA base modifications under conditions C3 and C4 (n=8 biological replicates). A detailed description of conditions can be found in the main *Figure 1*. Detailed information on RNA-modification-related genes can be found in *Table S19*.

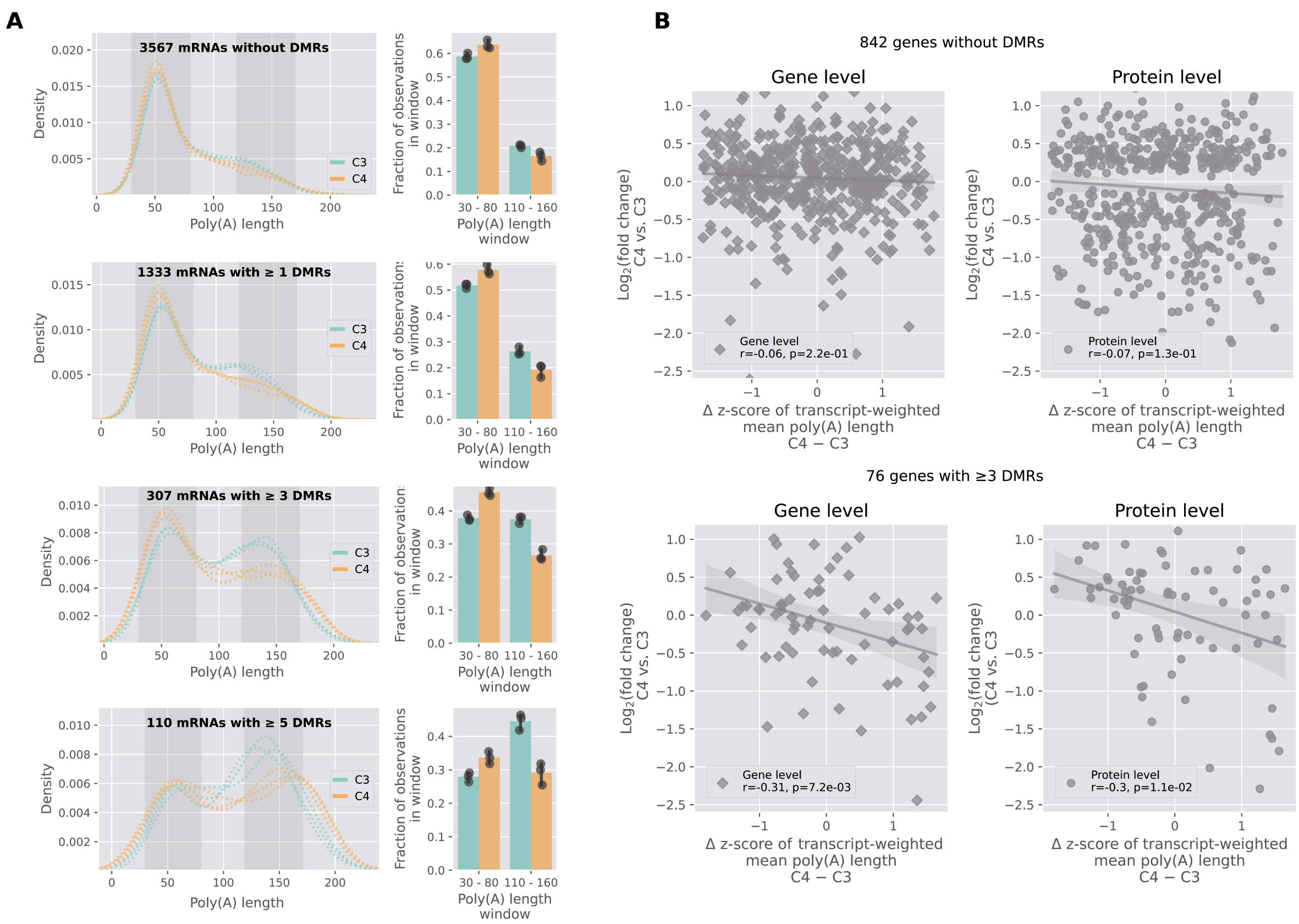

Figure S12: Poly(A)-tail length distributions in TGF-β1–treated (C3) and PDGF-BB/IL-1β–stimulated vascular smooth muscle cells (C4). (**A**) Kernel density plots of transcript-level poly(A) tail lengths across three replicates per condition, stratified by the number of differentially modified RNA bases (DMRs). Dotted lines indicate three replicates. Fractions of each replicate of observations in the predefined short-tail (30–80 nt) and long-tail (110–160 nt) windows are shown to the right. (**B**) Pearson correlation between all gene-level changes (nominally significant and non-significant) in z-score-normalized poly(A)-tail length (Δ, C4–C3) and $\log_2$ fold changes in gene or protein abundance. Transcript-level values were aggregated to the gene level.
